# Highly Reversible Supramolecular Light Switch for NIR Phosphorescence Resonance Energy Transfer

**DOI:** 10.1002/advs.202103041

**Published:** 2021-11-05

**Authors:** Conghui Wang, Xin‐Kun Ma, Peng Guo, Chunhui Jiang, Yao‐Hua Liu, Guoxing Liu, Xiufang Xu, Yu Liu

**Affiliations:** ^1^ College of Chemistry State Key Laboratory of Elemento‐Organic Chemistry Nankai University Tianjin 300071 China

**Keywords:** cell imaging, energy transfer, NIR, phosphorescence

## Abstract

Although purely organic room‐temperature phosphorescence (RTP) has drawn widespread attention in recent years, regulatable phosphorescence resonance energy transfer (PRET) supramolecular switch is still rare. Herein, single molecular dual‐fold supramolecular light switches, which are constructed by phenylpyridinium salts modified diarylethene derivatives (DTE‐Cn, *n* = 3, 5) and cucurbit[8]uril (CB[8]) are reported. Significantly, biaxial [3]pseudorotaxane displayed efficiently reversible RTP after binding with CB[8] and the phosphorescence quenching efficiency is calculated up to be 99%. Furthermore, the binary supramolecular assembly can coassemble with Cy5 to form ternary supramolecular assembly showing efficiently PRET, which is successfully applied in switchable near infrared (NIR) mitochondria‐targeted cell imaging and photocontrolled data encryption. This supramolecular strategy involving energy transfer provides a convenient approach for phosphorescent application in biology and material fields.

## Graphical abstract

A switchable supramolecular phosphorescence resonance energy transfer system for NIR cell imaging and data encryption is reported. This supramolecular strategy involving energy transfer provides a convenient approach for phosphorescent application in biology and material fields.

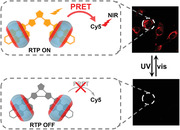

## 1 Introduction

Purely organic room‐temperature phosphorescence (RTP) has gained much attention recently for its distinct advantages, such as large Stokes shift, long‐lived lifetime, as well as the widespread applications.^[^ ^1^ ^]^ Many efforts have been devoted to exploring the strategy to induce RTP, among which “macrocyclic induce guest phosphorescence” is a noticeable approach to obtain effective RTP materials.^[^ ^2^, ^3^ ^]^ Tian et al. reported phosphor modified cyclodextrin derivatives immobilizing the phosphors through hydrogen bonding to emit efficient RTP emission.^[^ ^4^ ^]^ Recently, we reported cucurbit[n]uril‐mediated (*n* = 6–8) supramolecular phosphorescence assembly with long lifetime or high phosphorescence quantum yield.^[^ ^5^, ^6^, ^7^, ^8^ ^]^ However, controllable phosphorescence material remains a formidable challenge. It is well‐known that diarylethene derivatives could reversibly convert between two thermal stable isomers upon light irradiation.^[^ ^9^ ^]^ The excellent photochromic character and fatigue resistance endow them great potential to regulate the photonic and electronic properties, such as fluorescence,^[^ ^10^, ^11^, ^12^ ^]^ conductivity,^[^ ^13^, ^14^ ^]^ gas sorption,^[^ ^15^, ^16^ ^]^ the generation of ^1^O_2_,^[^ ^17^, ^18^, ^19^ ^]^ and mechanical motion,^[^ ^20^, ^21^ ^]^ based on the obviously different absorbance or geometrical structure of these two isomers. Vacha et al. reported a solid photoreversible RTP system based on energy transfer from a phosphorescence donor to a nonluminous photochromic diarylethene acceptor.^[^ ^22^ ^]^ However, in most systems, diarylethene play the role as “luminescence quencher” instead of “chromophore.”

In addition, redshift emissive systems become new tends in recent years and fluorescence energy transfer is a common strategy to obtain such system.^[^ ^23^ ^]^ Different from fluorescence energy transfer, phosphorescence resonance energy transfer (PRET) systems not only make the emission redshift, but transfer triplet energy to singlet state, overcoming the weakness of short lifetime for traditional acceptor dyes.^[^ ^24^, ^25^, ^26^, ^27^, ^28^ ^]^ George et al. realized “afterglow fluorescence” via PRET from organic phosphors to dyes in an amorphous polymer matrix.^[^ ^29^ ^]^ However, to our best knowledge, the system involving efficiently switchable NIR PRET in aqueous solution is not reported.

**Scheme 1:**
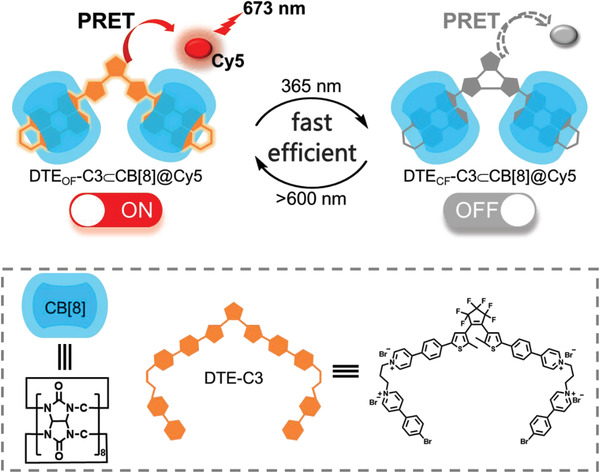
Schematic illustration and chemical structure of the photocontrolled NIR phosphorescence resonance energy transfer of DTE‐C3⊂CB[8]@Cy5.

Herein, we designed and synthesized two phenylpyridinium salts modified diarylethene derivatives (DTE‐Cn, *n* = 3, 5), in which 4‐(4‐(5‐methylthiophen‐2‐yl) phenyl) pyridinium was linked with 4‐(4‐bromophenyl)‐pyridinium by propyl (*n* = 3, DTE‐C3) or amyl (*n* = 5, DTE‐C5). Interestingly, the different binding formation led to different emission behaviours: cucurbit[7]uril (CB[7]) tended to separate the two phenylpyridinium salts leading to RTP quench; while the complexation with CB[8] could form unique “single molecular dual‐fold” and this formation could reduce the distance between two phenylpyridinium salts inducing the efficient RTP emission. Notably, the DTE‐C3⊂CB[8] as phosphorescent donor could transfer energy to NIR‐fluorescent dye (Cy5) acceptor and the PRET process could be tuned reversibly (**Scheme** **1**). In addition, this switchable PRET system was further applied in targeted cell imaging and information encryption.

## 2 Results and Discussion

**Figure 1:**
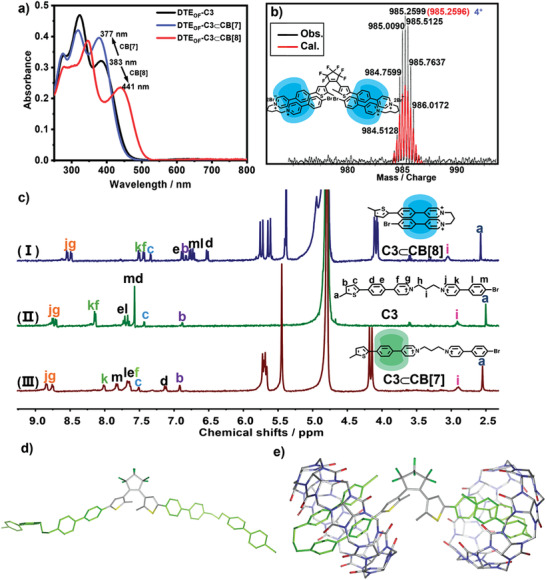
a) UV–vis spectral changes of DTE‐C3 (8 × 10^−6^ m) upon adding 2.0 equivalent CB[7] and CB[8]. b) High‐resolution electrospray ionization mass spectrum of DTE‐C5⊂CB[8]. c) ^1^H NMR spectral changes of (II) C3 after adding 1.0 equivalent (I) CB[8] and (III) CB[7]. (400 MHz, D_2_O, 298 K). d) The proposed model for DTE‐C3. e) The proposed model for DTE‐C3 ⊂CB[8].

The synthetic route and compound characterization of diarylethene derivatives DTE‐Cn (*n* = 3, 5) and reference compounds Cn (*n* = 3, 5) were shown in Schemes S1–S5 and Figures S35–S49 (Supporting Information). The host–guest binding stoichiometry was determined to be 2:1 for both DTE‐Cn⊂CB[7] and DTE‐Cn⊂CB[8] according to the Job's plot (Figure S1, Supporting Information). Though DTE‐Cn possessed the same binding stoichiometry with CB[7] and CB[8], the UV–vis titration spectra displayed totally different results, which suggested that these assembly may have the different binding formation. The absorption of DTE‐Cn centered around 380 nm shifted to long wave region with decreased absorbance upon adding 2.0 equivalent of CB[8], while the absorption showed hypsochromic shift with enhanced absorbance upon adding 2.0 equivalent of CB[7] (**Figure** **1**a; and Figure S2, Supporting Information). The large bathochromic shift can be ascribed to the CB[8]‐stabilized charge transfer (CT) interaction, which was somewhat diminished when CB[7] was attached. Two clear isosbestic points appeared upon continuous adding CB[8] to the solution of DTE_OF_‐Cn and the binding constant (*K*_s_) were accordingly calculated as 7.96 × 10^12^ m ^−2^ and 2.09 × 10^12^ m ^−2^, respectively, and these high binding affinity ensured the strong complexation between DTE‐Cn and CB[8] (Figure S3, Supporting Information).

It was reported that CB[8] with a large cavity could accommodate two pyridinium guests simultaneously to form 1:2 supramolecular assembly, and “Donor–Acceptor” guest molecule linked with a long and flexible linker, such as propyl or amyl, could form 1:1 assembly with CB[8] through host‐stabilized intramolecular CT interaction.^[^ ^30^ ^]^ As comparison, CB[7] possessing a smaller cavity than CB[8], could only accommodate one pyridinium guest to form 1:1 supramolecular complex.^[^ ^31^, ^32^ ^]^ Hence, it was anticipated that CB[8] could induce DTE‐Cn self‐fold within its cavity through host‐stabilized intramolecular CT interaction, while CB[7] could separate two pyridinium moieties and weaken CT interaction between the donor and acceptor, which led to the folded configuration was unable to form.

The mass spectra gave the apparent evidence for the self‐fold supramolecular assembly of DTE‐Cn⊂CB[8] (Figure 1b; and Figure S4, Supporting Information). For example, the parent peak of [DTE‐C5 + 2CB[8] – 4Br^−^]^4+^ corresponding to the 2:1 host–guest complex was found in mass spectrometry, which matched the calculated result very well and excluded the possibility of “2:2” bonding formation. The morphological information of DTE_OF_‐Cn⊂CB[8] revealed from transmission electron microscope (TEM) images showed the nanosheet instead of nanofiber, which further excluded the possibility of liner assembly (Figure S5, Supporting Information). To further confirm the folding conformation of DTE‐Cn⊂CB[8], NMR experiments were performed. However, considering the poor solubility of DTE‐Cn at deuterium oxide (≤ 10 × 10^−6^ m), the reference compounds (Cn, *n* = 3, 5) without hexafluorocyclopentene were synthesized to investigate the binding behaviors. The absorbance of Cn showed the apparent bathochromic shift upon adding CB[8] and the binding stoichiometry was determined to be 1:1 (Figure S6, Supporting Information). Mass spectrum also gave the existence of binary [Cn + CB[8] – 2Br^−^]^2+^ complexes (Figure S7, Supporting Information). All these results were similar with DTE‐Cn⊂CB[8], suggesting that DTE‐Cn and Cn possessed the similar binding conformation upon complexing with CB[8]. The addition of CB[8] led to aromatic protons showing obvious upfield shifts, while the ones of methyl and alkyl chain part gave downfield shifts, which implied the phenylpyridinium moieties were included inside the cavity of CB[8], while methyl and alkyl chain located outside the CB[8] cavity (Figure 1c; and Figure S8, Supporting Information). Moreover, CB[8] portal protons split into two sets of equivalent peaks after complexing with Cn, demonstrating the centrosymmetric geometry of CB[8] had been broken.^[^ ^33^ ^]^ Diffusion Ordered Spectroscopy (DOSY) of C5 and C5⊂CB[8] solution gave the diffusion coefficient of 4.63 × 10^−10^ m^2^ s^−1^ and 2.65 × 10^−10^ m^2^ s^−1^, respectively, which were in consistent with the reported result (Figure S9, Supporting Information).^[^ ^34^, ^35^ ^]^ 2D ^1^H‐^1^H ROESY spectra also showed significant NOE cross peaks between two pyridinium groups, providing reliable evidence for the folding configuration (Figure S10, Supporting Information). Furthermore, simulated structure by density functional theory provided visible image of “molecular folding” conformation (Figure 1d,e). Combined with these results, we could conclude that Cn was induced into the cavity of CB[8] with folding configuration, which further suggested that DTE‐Cn could be included in CB[8] cavity to form 2:1 complex with the same folded configuration of Cn.

The UV–vis, Job's plot and mass spectra of Cn⊂CB[7] were also in accordance with DTE‐Cn⊂CB[7] (Figures S11 and S12, Supporting Information). Very interestingly, upon adding 1.0 equivalent of CB[7] to the solution of C5, alkyl chain and pyridinium parts showed upfield shifts while other aromatic protons displayed downfield shifts, indicating CB[7] resided over the alkyl chain moieties (Figure S8, Supporting Information). However, the chemical shifts were quite different when adding 1.0 equivalent of CB[7] to the solution of C3. The protons of phenylpyridinium in donor (*H*_f_) displayed larger upfield shifts than the protons in acceptor (*H*_k_), indicated that CB[7] was prefer to be positioned over 4‐(4‐(5‐methylthiophen‐2‐yl)phenyl)pyridinium moiety (Figure 1c; and Figure S13, Supporting Information). For C5, donor and acceptor both provided positive charge for each portal of the CB[7] and the ion‐dipole interaction could stabilize the formed [2]pseudorotaxanes.^[^ ^30^ ^]^ But, the alkyl chain of C3 was shorter than C5 so that CB[7] translocated to the more hydrophobic donor moiety to form the 1:1 host–guest complexes.

**Figure 2:**
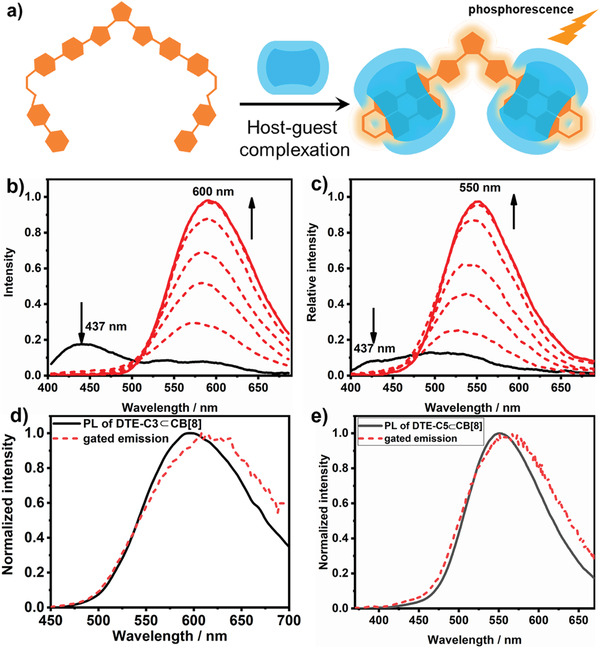
a) Schematic illustration of host–guest complexation. Photoluminescence spectral changes of b) DTE_OF_‐C3 and c) DTE_OF_‐C5 after adding 0.0–2.0 equivalent CB[8] ([DTE_OF_‐C3] = 8 × 10^−6^ m, *λ*_ex_ = 360 nm, in 1%MeOH‐H_2_O; [DTE_OF_‐C5] = 8 × 10^−6^ m, *λ*_ex_ = 350 nm, in H_2_O). Normalized photoluminescence spectrum and phosphorescent spectrum of d) DTE_OF_‐C3⊂CB[8] and e) DTE_OF_‐C5⊂CB[8] (delayed time of 50 µs).

After validating the binding conformation of DTE‐Cn with CB[7] and CB[8], we further explored the photoluminescence behavior. The excitation spectra of DTE_OF_‐Cn⊂CB[7] were similar to that of DTE_OF_‐Cn (Figure S14, Supporting Information). The emission only showed dramatic enhancement and the red band emission displayed hypsochromic shift after complexing with CB[7] (Figure S15, Supporting Information). The lifetime of DTE_OF_‐C5⊂CB[7] was measured to be 1.52 ns, which was slightly higher than that of DTE_OF_‐C5 (1.14 ns) due to the encapsulation of CB[7] prevented the nonradiative relaxation and avoided quenchers in the solution (Figure S16, Supporting Information). The lifetime of DTE_OF_‐C3⊂CB[7] (1.53 ns) was also higher than that of DTE_OF_‐C3 (1.26 ns) (Figure S16, Supporting Information). As for DTE_OF_‐Cn⊂CB[8], a new excitation peak appeared at 400–500 nm, which was attributed to its CT characteristic (Figure S14, Supporting Information). Different from DTE_OF_‐Cn⊂CB[7], the emission peak around 437 nm disappeared after continuously adding CB[8] to the solution of DTE_OF_‐Cn and a emission peak emerged at 600 nm for DTE‐C3⊂CB[8] and 551 nm for DTE‐C5⊂CB[8] at the same time, resulting from the formation of folded configuration and enhanced CT interaction (**Figure** **2**a–c). The difference between emission wavelength for DTE_OF_‐Cn⊂CB[8] was because of the length of alkyl spacer segment which influenced the degree of *π*–*π* stacking.^[^ ^36^ ^]^ More interestingly, these emission peak were confirmed as phosphorescence for the following reasons. First, varied temperature experiment revealed that the emission intensity decreased with increasing temperature, which was the typical feature of phosphorescence (Figure S17, Supporting Information).^[^ ^37^ ^]^ Second, the gated emission spectra were consistent with photoluminescence spectra, which also verified its phosphorescent property (Figure 2d,e). Third, the lifetime of DTE_OF_‐Cn⊂CB[8] were prolonged to microsecond scale after complexing with CB[8] (Figure S18, Supporting Information). Besides, DTE_OF_‐Cn didn't show apparent RTP signal (Figure S19, Supporting Information). The efficient phosphorescence resulted from unique molecular folding structure: the introduction of heteroatom (bromine) not only played the acceptor role in process of intramolecular CT but also increase the rate of intersystem crossing through intramolecular halogen bond.^[^ ^36^ ^]^ Furthermore, CB[8] could suppress nonradiative decay rates by encapsulating the guest molecular within its cavity.^[^ ^38^, ^39^ ^]^

Given that the backbone of diarylethene could undergo reversible conversion between two thermal stable isomers (DTE_OF_‐Cn and DTE_CF_‐Cn), the photoinduced photochromic performances were investigated. After continuously irradiating with 365 nm UV light, the color of the solution containing DTE‐Cn changed from colorless to blue (Figure S20, Supporting Information). Meanwhile a new absorption peak around 640 nm appeared due to the DTE_CF_‐Cn was generated. In addition, irradiated with > 600 nm visible light the UV–vis spectra and the color of solution recovered to the initial state (Figure S20, Supporting Information). Upon alternating UV and visible light irradiation this process could be repeated several times with no apparent photochemical damage indicating the good fatigue resistance (Figure S21, Supporting Information). The conversion efficiency of DTE‐Cn was determined to be above 95% by NMR integral ratio changes, suggesting the excellent photochromic ability (Figure S22, Supporting Information). Furthermore, the UV–vis spectra of DTE‐Cn⊂CB[7] and DTE‐Cn⊂CB[8] upon 365 and > 600 nm light irradiation were also measured showing excellent photochromic characteristics. (Figures S23 and S24, Supporting Information).

**Figure 3:**
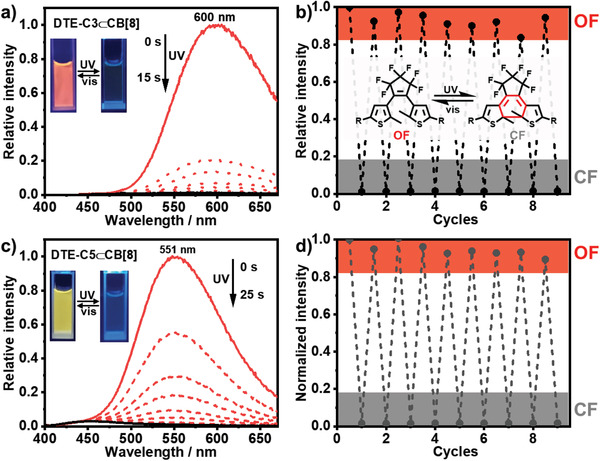
Photoluminescence spectral changes of a) DTE_OF_‐C3⊂CB[7] and c) DTE_OF_‐C5⊂CB[8] after 365 nm light irradiation. Photoluminescence fatigue resistance of b) DTE‐C3⊂CB[8] and d) DTE‐C5⊂CB[8] upon alternating UV and visible light irradiation. ([DTE‐C3] = [DTE‐C5] = 8 × 10^−6^ m, [CB[7]] = [CB[8]] = 16 × 10^−6^ m).

The formation of DTE_CF_‐Cn with the enlargement of *π*‐electron delocalization under UV light irradiation could also be used to regulate the emission behaviors. Hence, the photoinduced changes in photoluminescence spectra were investigated at room temperature. As shown in Figure S25 (Supporting Information), the emission around 500 nm of DTE_OF_‐Cn⊂CB[7] gradually decreased after 365 nm light irradiation, while the emission at 437 nm unchanged. The fluorescence of DTE_OF_‐Cn⊂CB[7] could recover to initial state after > 600 nm light irradiation (Figure S25, Supporting Information). As for DTE_OF_‐Cn⊂CB[8], the emission gradually quenched upon 365 nm light irradiation and the quenching efficiency was calculated to be 99% by comparing the emission intensity before and after 365 nm UV irradiation (**Figure** **3**a,c). Subsequently, the emission intensity recovered to initial level upon continuous irradiation by > 600 nm visible light and this process could be repeated several times with no apparent fading implying excellent photochromic performance (Figure 3b,d; and Figure S26, Supporting Information). The phenomenon observed from phosphorescence spectrum was similar (Figure S27, Supporting Information).

**Figure 4:**
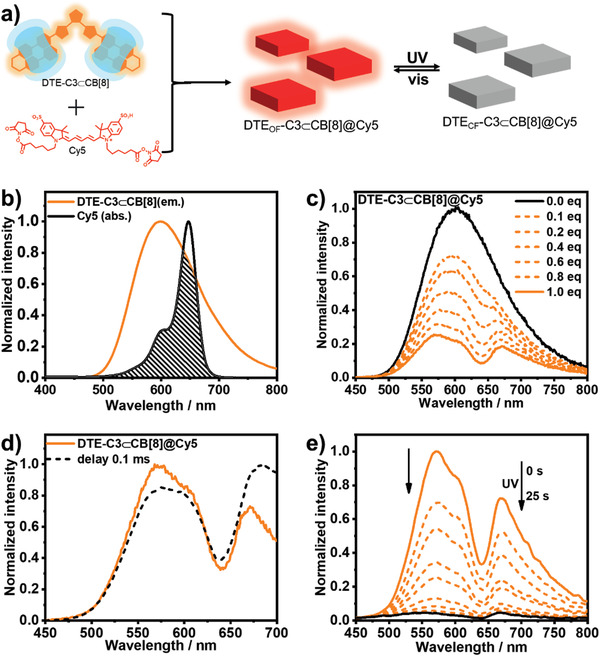
a) Schematic illustration of the phosphorescence energy transfer process. b) Spectral overlap between the absorption of Cy5 and the emission of DTE_OF_‐C3⊂CB[8]. c) Photoluminescence spectral changes of DTE_OF_‐C3⊂CB[8] upon continuous adding Cy5. [DTE‐C3] = 20 × 10^−6^ m, [CB[8]] = 40 × 10^−6^ m, in 1%MeOH‐H_2_O. d) Phosphorescence and photoluminescence spectrum of DTE_OF_‐C3⊂CB[8]@Cy5 (delayed time of 0.1 ms). e) Photoluminescence spectral changes of DTE_OF_‐C3⊂CB[8]@Cy5 upon continuous irradiation with 365 nm UV light. [DTE‐C3] = [Cy5] = 20 × 10^−6^ m, [CB[8]] = 40 × 10^−6^ m.

A commercially available NIR dye (Cy5) was loaded to the supramolecular phosphorescence switch DTE_OF_‐C3⊂CB[8] to realize the NIR phosphorescence energy transfer (**Figure** **4**a). As shown in Figure 4b, the absorption of Cy5 matched perfectly with the emission of DTE_OF_‐C3⊂CB[8], which was the prerequisite for energy transfer. The Tyndall effect and scanning electron microscope (SEM) images showed that the assembly DTE‐C3⊂CB[8]@Cy5 existed as sheet nanoparticles (Figure S28, Supporting Information). The formation of supramolecular nanoparticle provided suitable distance for energy transfer. The NMR spectrum of reference compound C3⊂CB[8] did not show apparent change after adding Cy5 indicating that the addition of Cy5 did not disrupt the phosphorescence switch, while Cy5 showed slight downfield shifts, implied that Cy5 was tightly encapsulated (Figure S29, Supporting Information). After satisfying the conditions of spectral overlap and distance, as expected, the emission intensity at 600 nm decreased with the gradual addition of Cy5 accompanied with a new peak emerged at 673 nm (Figure 4c). The gated emission and lifetime of DTE_OF_‐C3⊂CB[8]@Cy5 implied that the emission at 673 nm was delayed‐fluorescence (Figure 4d; and Figure S30, Supporting Information). Furthermore, the emission intensity at 673 nm increased with the decreased temperature excluding the possibility of thermally activated delayed fluorescence (Figure S31, Supporting Information). The phosphorescence lifetime at 600 nm of DTE_OF_‐C3⊂CB[8] decreased to 2.24 µs with the addition of Cy5 indicated the phosphorescence energy transfer from triplet to singlet (Figure S30, Supporting Information). The solution of CB[8]@Cy5 only showed the very weak fluorescence suggesting the efficient energy transfer process (Figure S32, Supporting Information). The calculated energy transfer efficiency was 28%.^[^ ^28^ ^]^ This energy transfer process could also be regulated by light with different wavelength, that was when irradiated with 365 nm UV light, the emission intensity and lifetime decreased because of the generation of closed form diarylethene DTE_CF_‐C3 (Figure 4e; and Figure S30, Supporting Information), and the emission intensity could recover to initial state by irradiating > 600 nm visible light (Figure S33, Supporting Information). The emission intensity of CB[8]@Cy5 remained unchanged under the same UV light irradiation, indicating that the switchable energy transfer resulted from the photochromism of diarylethene (Figure S34, Supporting Information).

**Figure 5:**
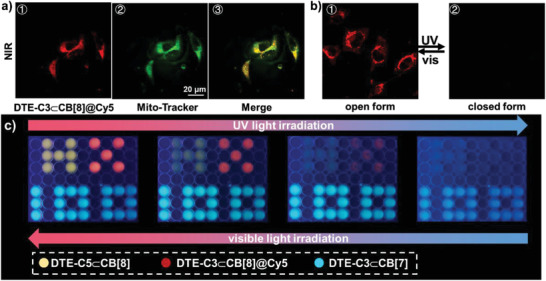
a) Laser confocal images of A549 cells costained with Mito‐Tracker Green, DTE_OF_‐C3⊂CB[8]@Cy5, and merged image. The excitation wavelength was set as 405 nm and the emission was collected from 650 to 750 nm for DTE‐C3⊂CB[8]@Cy5. [DTE‐C3] = [Cy5] = 10 × 10^−6^ m, [CB[8]] = 20 × 10^−6^ m. b) Laser confocal images of DTE‐C3⊂CB[8]@Cy5 before and after 365 nm light irradiation. c) Schematic illustration of the application for information encryption.

Benefiting from the photocontrolled NIR energy transfer system, we further carried out cell imaging experiment. Confocal laser scanning microscopy of A549 cells incubated with DTE_OF_‐C3⊂CB[8]@Cy5 suggested that the assemblies could be easily internalized into the cell and displayed good colocalization with the mitochondria marker Mitotracker Green (**Figure** **5**a). Furthermore, the cell imaging could also be reversibly regulated by light (Figure 5b). Considering the obvious difference in photochromic rate and color changes of phosphorescence or fluorescence between these supramolecular complexes, a photocontrolled digital display for information encryption was fabricated. As showed in Figure 5c, the digital pattern “HX100” was constructed with different complexes. The photochromic rates followed the order of DTE‐Cn⊂CB[8] > DTE‐Cn⊂CB[7]. When irradiated with 365 nm UV light, the “H” and “X” disappeared immediately for the fast conversion from DTE_OF_‐Cn⊂CB[8] to DTE_CF_‐Cn⊂CB[8]. Subsequently, the color of “100” changed from cyan to blue with the prolonged 365 nm UV light irradiation. This process not only realized the change of digital pattern, for example from “HX100” to “100”, but also accompanied with the change of color, such as from cyan to blue. Besides, the color and digital pattern could also recover to initial state by irradiating with > 600 nm visible light to achieve the information encryption and anticounterfeiting.

## 3 Discussion and Results

In conclusion, we synthesized and fabricated binary supramolecular phosphorescence switches composed of diarylethene derivatives DTE‐Cn (*n* = 3, 5) and CB[8]. CB[8] could induced guest molecular into the cavity with folded configuration and promoted intramolecular charge transfer leading to efficient RTP emission. As comparison, the complexation with CB[7] could only construct fluorescence switches due to the folding configuration were unable to form. These photophysical properties could be reversibly modulated by the photochromism of diarylethene moiety with high quenching efficiency. Interestingly, the phosphorescence switch as donor could transfer triplet energy to a NIR fluorescence dye Cy5 achieving photocontrolled NIR delayed fluorescence. Besides, the application for NIR cell imaging and information encryption was also demonstrated. This work may have great potential on the design and fabricate intelligent materials with versatile optical properties.

## 4 Experimental Section

### Reagents and Materials

All starting materials were obtained commercially as analytical‐grade and used without further purification, unless otherwise noted. NMR spectra were recorded on a Bruker AV400 spectrometer at 25 °C, DOSY spectra were measured on a zhongke‐Oxford I‐400 instrument. High‐resolution MS was performed on a Q‐TOF LC‐MS in electrospray ionization mode. Absorption spectra were recorded on a Thermo Fisher Scientific EVO300 PC spectrophotometer in a conventional rectangular quartz cell (10 × 10 × 45 mm) at 25 °C. Photoluminescence spectra, quantum yields and lifetimes were measured by means of time‐correlated single photon counting on FLS980 and FS5 instrument (Edinburg Instruments, Livingstone, UK). TEM experiments were carried out on an FEI Tecnai G2 F20 microscope operating at 200 KV. SEM images were recorded on a JEOL JSM‐7500F scanning electronic microscope operating at an accelerating voltage of 30 keV. The UV light irradiation experiments were carried out using a ZF‐7A lamp (*λ* = 365 nm, 8 W), and the visible light irradiation experiment (*λ* > 600 nm) was carried out using a CEL4HXF300 14V 50W xenon lamp with cutoff filter.

### Cell Imaging

The cancer cell line including human lung adenocarcinoma cells (A549) were obtained from the Cell Resource Center of China Academy of Medical Science (Beijing, China). The well‐cultured cells were incubated with DTE‐C3⊂CB[8]@Cy5 ([DTE‐C3] = [Cy5] = 10 × 10^−6^ m, [CB[8]] = 20 × 10^−6^ m) for 10 h. The cells were then washed with PBS and stained with MitoTracker Green at 310 K for 30 min. Then the cells were further washed three times with phosphate buffer solution, fixed with 4% paraformaldehyde for 15 min, and then observed with a confocal microscope.

## Conflict of Interest

The authors declare no conflict of interest.

## Supporting information

Supporting Information

## Data Availability

The data that support the findings of this study are available from the corresponding author upon reasonable request.
