# Supplementary material for: Highly Reversible Supramolecular Light Switch for NIR Phosphorescence Resonance Energy Transfer
Source: Adv Sci (Weinh). 2021 Nov 5;9(2):2103041. doi: 10.1002/advs.202103041 (PMC8805551; doi:10.1002/advs.202103041)
Supplement: Supplementary file 1 — Supporting Information [file ADVS-9-2103041-s001.pdf]

© 2021 Wiley-VCH GmbH

ADVANCED SCIENCE
Open Access

Supporting Information

for *Adv. Sci.,* DOI: 10.1002/advs.202103041

Highly Reversible Supramolecular Light Switch for NIR Phosphorescence Resonance Energy Transfer

*Conghui Wang, Xin-Kun Ma, Peng Guo, Chunhui Jiang, Yao-Hua Liu, Guoxing Liu, Xiufang Xu and Yu Liu **

Supporting Information

Highly Reversible Supramolecular Light Switch for NIR Phosphorescence Resonance Energy Transfer

*Conghui Wang, Xin-Kun Ma, Peng Guo, Chunhui Jiang, Yao-Hua Liu, Guoxing Liu, Xiufang Xu and Yu Liu**

College of Chemistry, State Key Laboratory of Elemento-Organic Chemistry, Nankai University, Tianjin 300071, China

*E-mail: yuliu@nankai.edu.cn

Supporting Information

1. Characterization of Assembly Behavior 1
2. Characterization of Luminous Properties 10
3. Characterization of Photochromic Performance 13
4. Measurement for Quantum Yield and Lifetime 23
5. Synthetic Routes and Characterization of Compound 24
6. Computational details 38
7. Reference 38

1. Characterization of Assembly Behavior

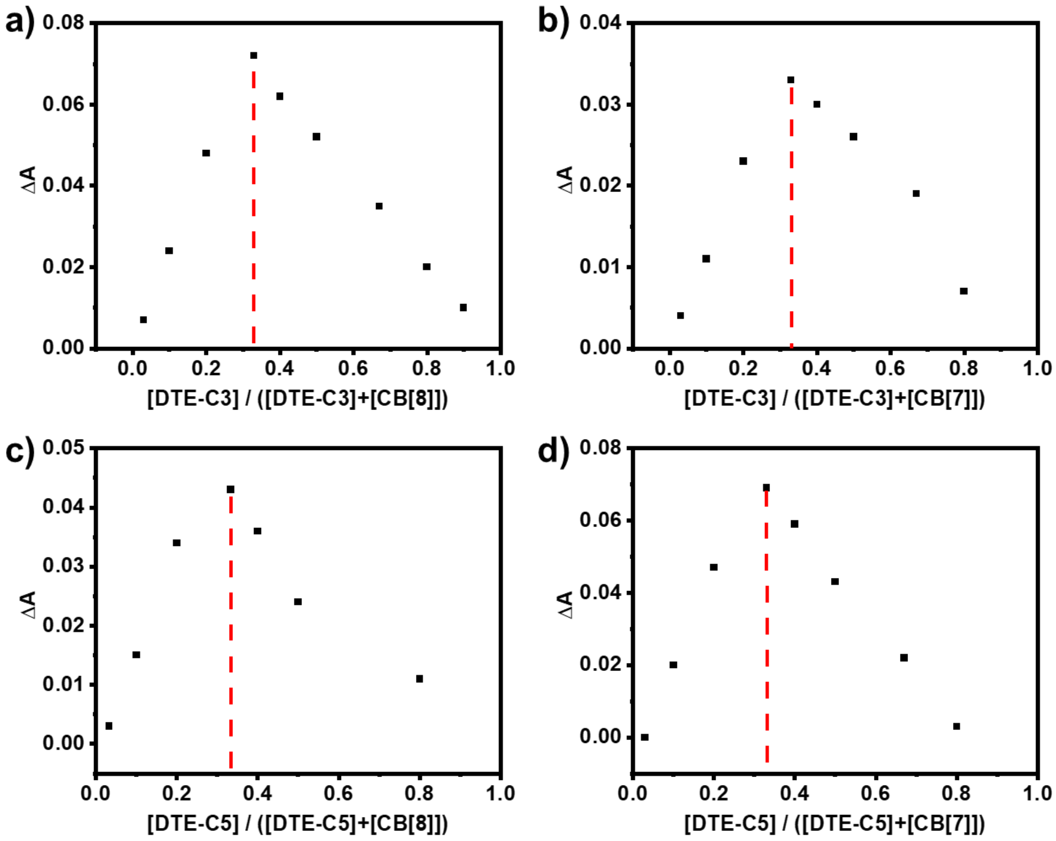

Figure S1. Job's plot of CB[7]/CB[8] and DTE_{OF}-Cn ([CB[7]] + [DTE_{OF}-Cn] = 10 μM, [CB[8]] + [DTE_{OF}-Cn] = 10 μM, n = 3, 5).

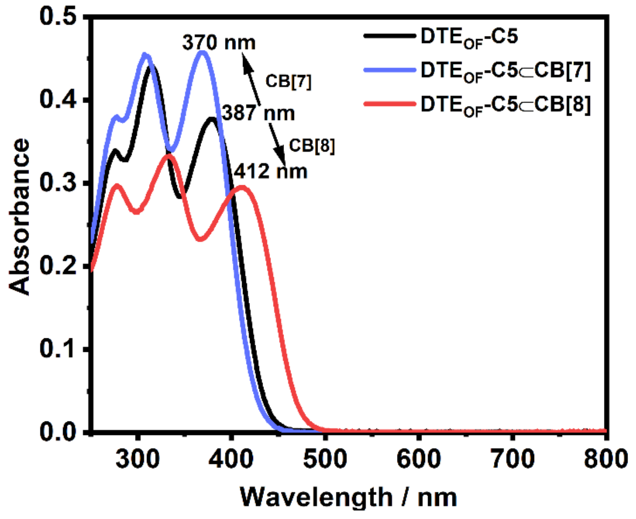

Figure S2. UV/vis spectral changes of DTE-C5 (8 μM) upon adding 2.0 equivalent CB[7] and CB[8].

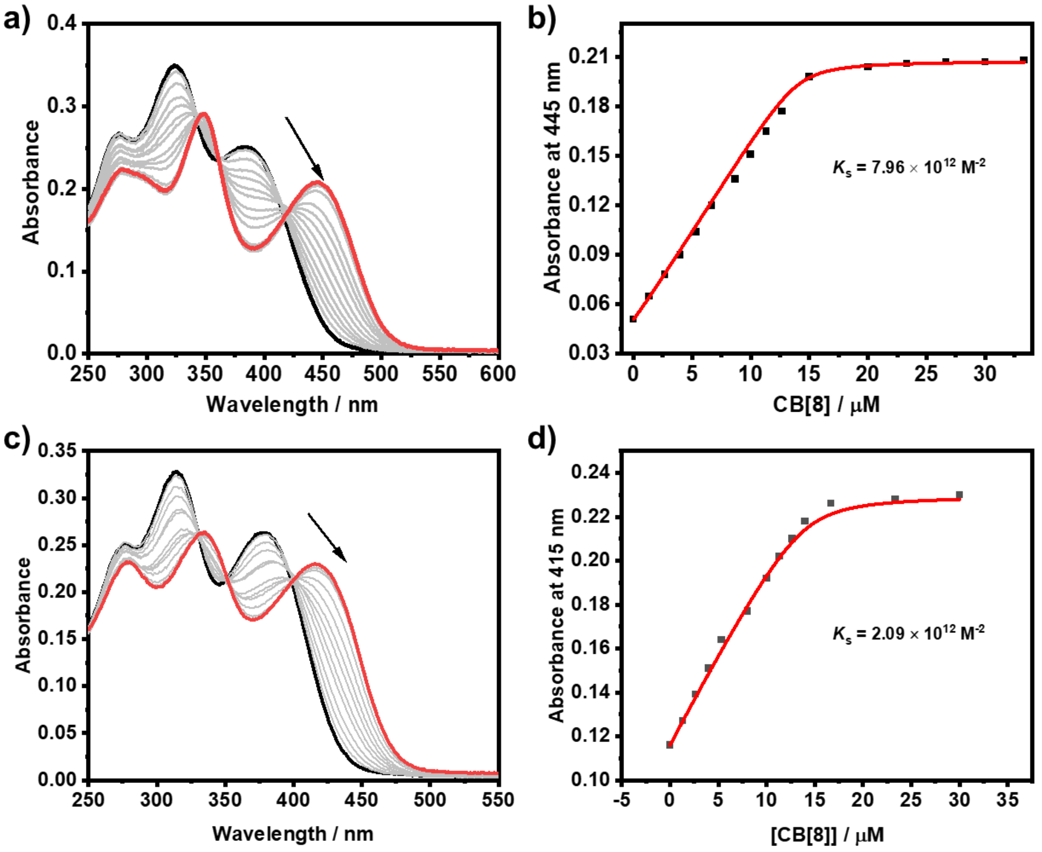

Figure S3. a) UV/vis titration spectrum of DTE_{OF}-C3 (6.7 μM) upon addition of CB[8] (0.0 - 33.3 μM). b) Nonlinear least-squares analysis of the differential absorbance at 445 nm to calculate the K_S value. c) UV/vis titration spectrum of DTE_{OF}-C5 (6.7 μM) upon addition of CB[8] (0.0 - 30.0 μM). d) Nonlinear least-squares analysis of the differential absorbance at 415 nm to calculate the K_S value.

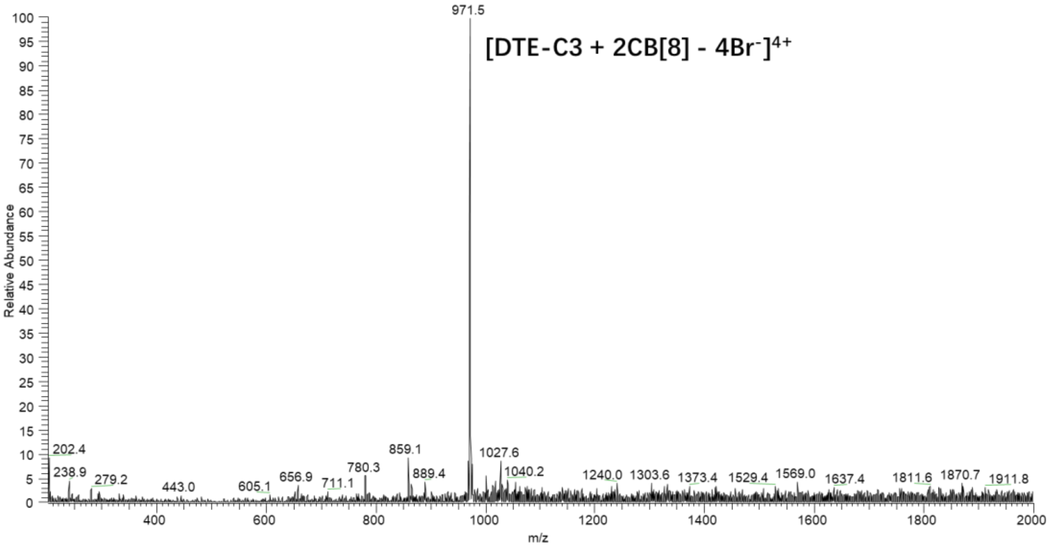

Figure S4. ESI-MS of DTE-C3⊂CB[8].

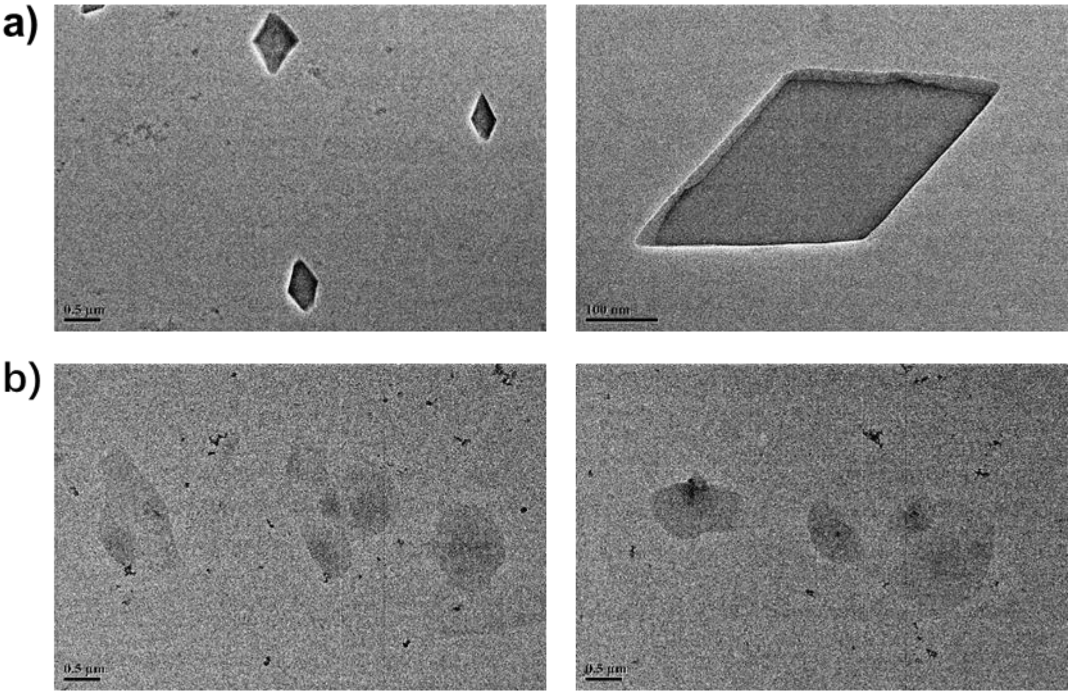

Figure S5. TEM images of a) DTE_{OF}-C5⊂CB[8] and b) DTE_{OF}-C3⊂CB[8]. The aqueous solution used for TEM measurements contains DTE-Cn (8 μM) and CB[8] (16 μM).

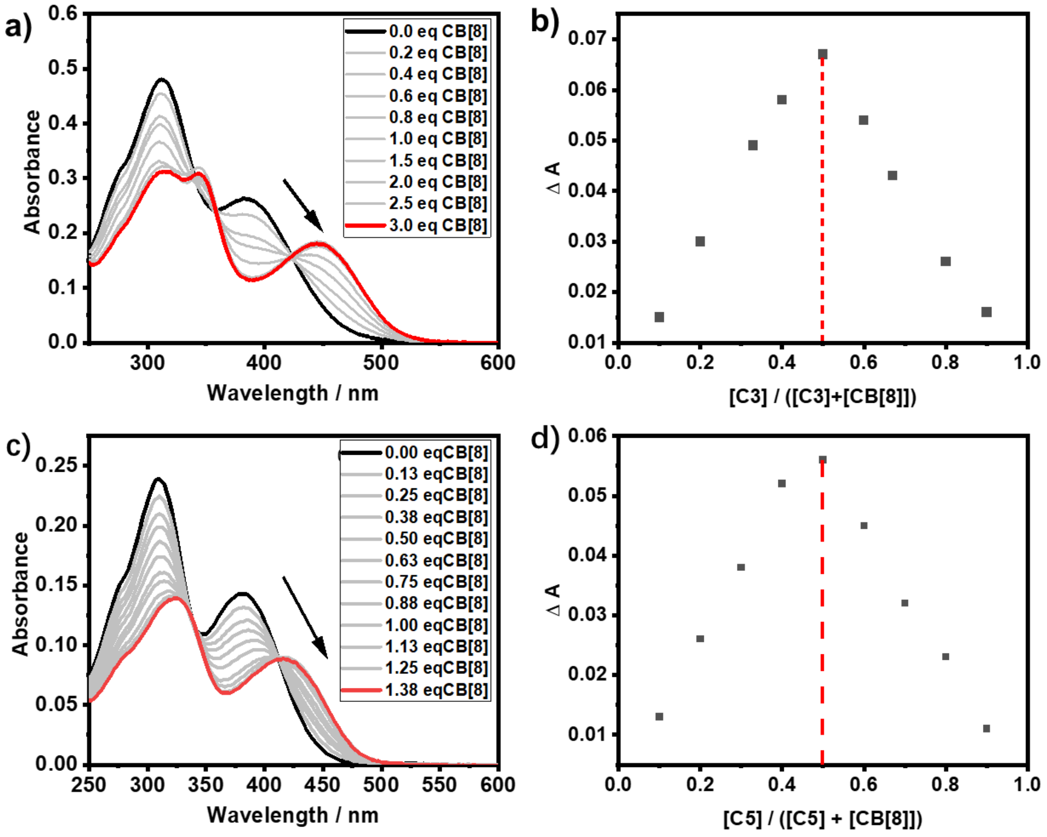

Figure S6. UV/vis spectral changes of a) C3 and c) C5 upon adding CB[8]. ([C3] = 20 μM, [C5] = 10 μM, in H_2O) b) Job's plot of CB[8] and C3 ([CB[8]] + [C3] = 20 μM). d) Job's plot of CB[8] and C5 ([CB[8]] + [C5] = 20 μM).

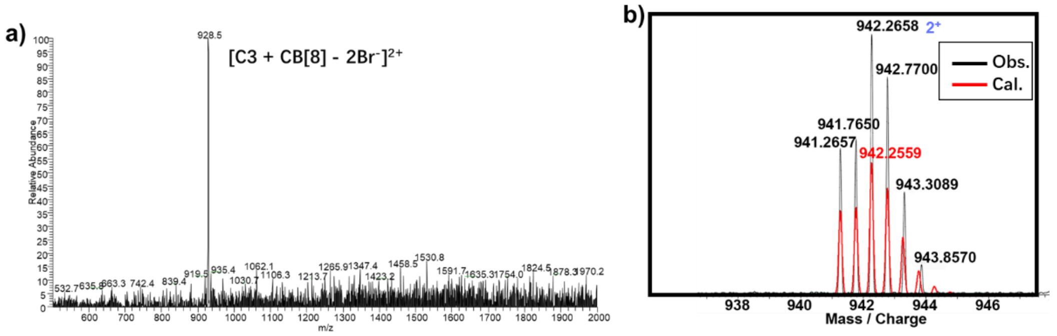

Figure S7. ESI-MS of a) C3⊂CB[8] and b) C5⊂CB[8].

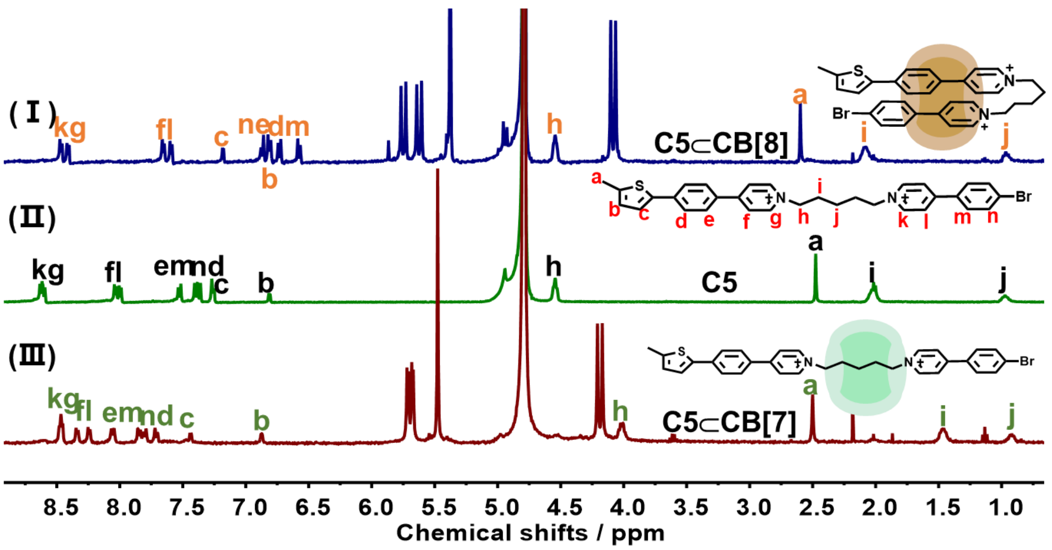

Figure S8. ^{1}H NMR spectral changes of (Ⅱ) C5 after adding 1.0 equivalent (Ⅰ) CB[8] and (Ⅲ) CB[7] (400 MHz, D_2O, 298 K).

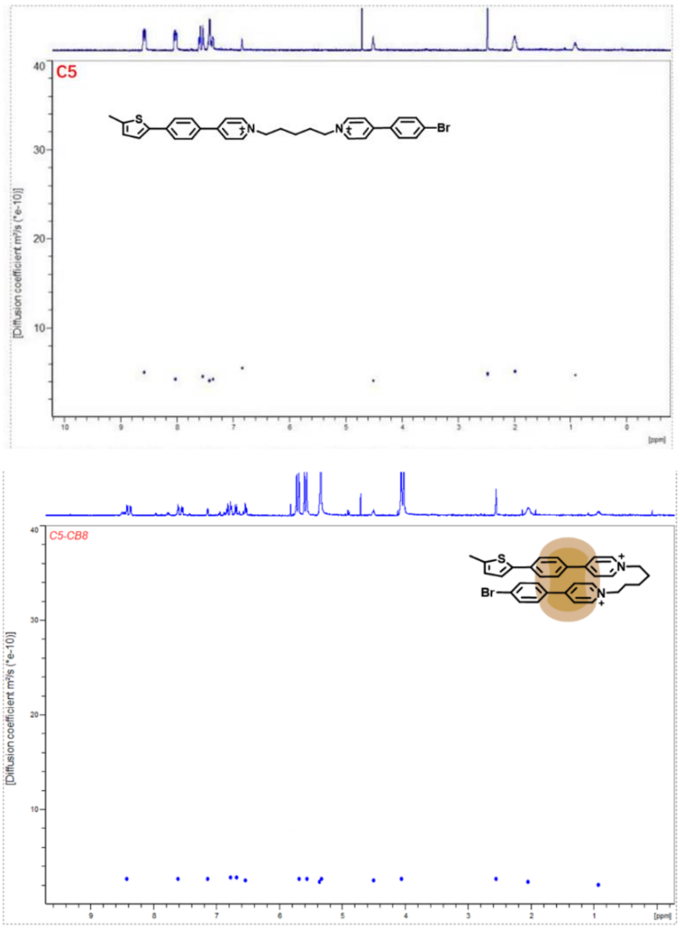

Figure S9. 2D Diffusion-ordered NMR spectrum of C5 and C5⊂CB[8]. ([C5] = [CB[8]] = 0.5 mM, D_2O, 298 K)

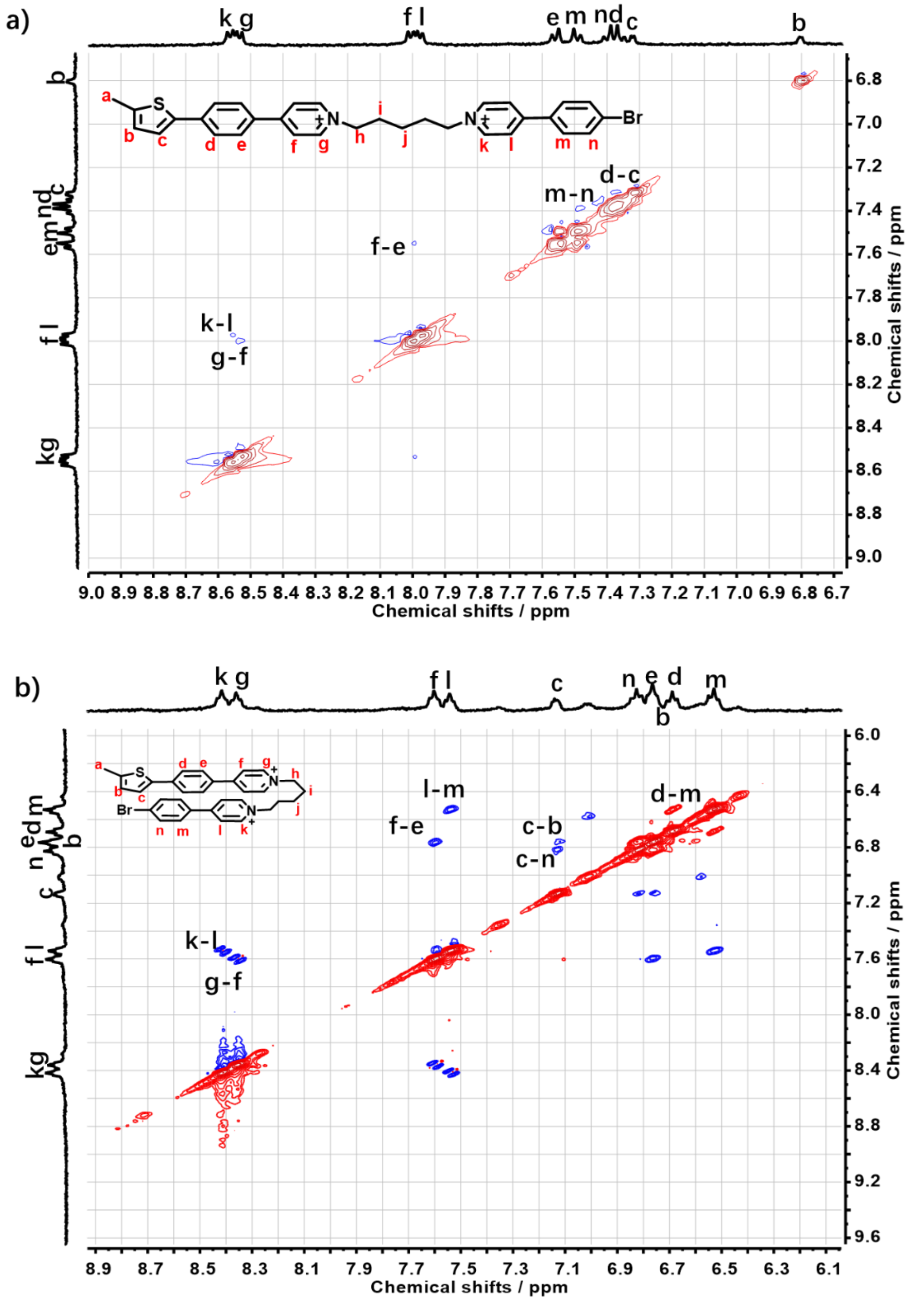

Figure S10. a) 2D ^{1}H-^{1}H COSY of C5. b) ROESY of C5⊂CB[8]. (D_2O, 298 K)

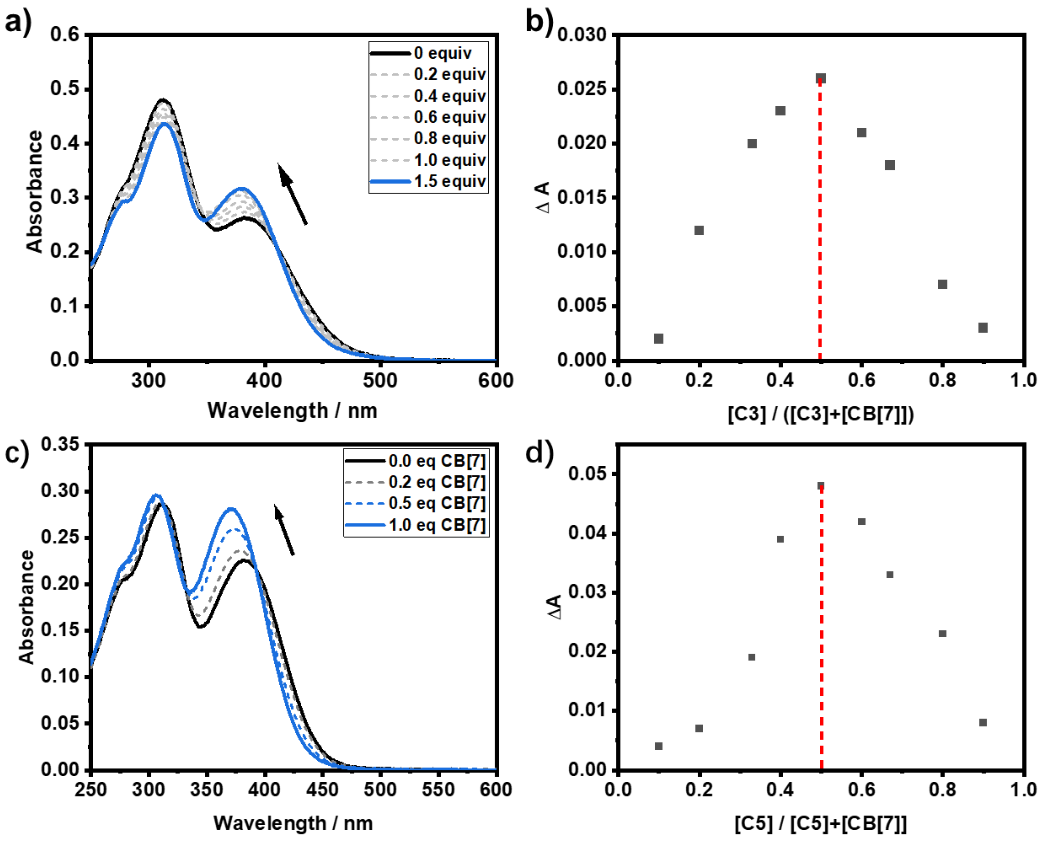

Figure S11. UV/vis spectral changes of a) C3 and c) C5 upon adding CB[7]. ([C3] = [C5] = 20 μM, in H_2O) b) Job's plot of CB[7] and C3 ([CB[7]] + [C3] = 20 μM). d) Job's plot of CB[7] and C5 ([CB[7]] + [C5] = 20 μM).

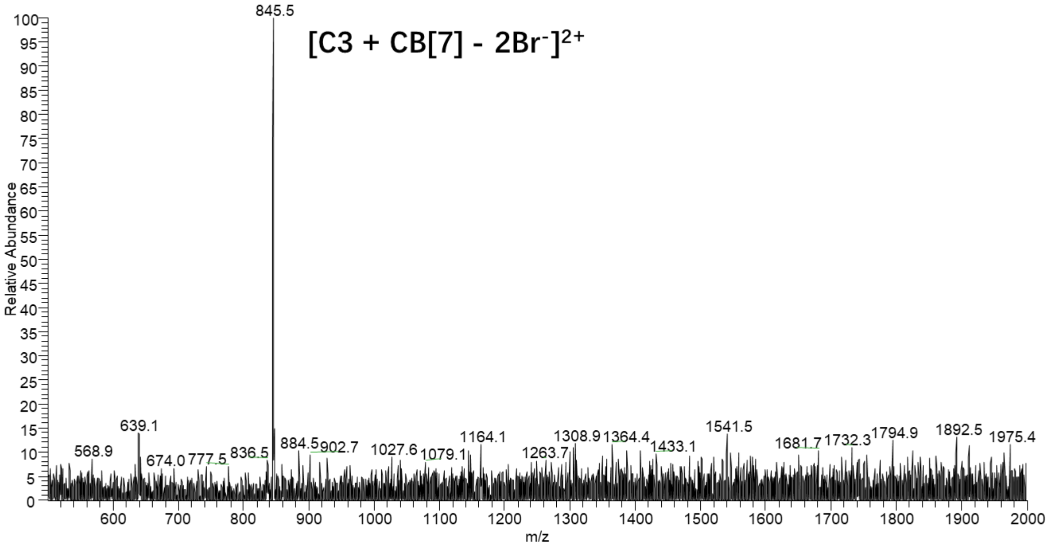

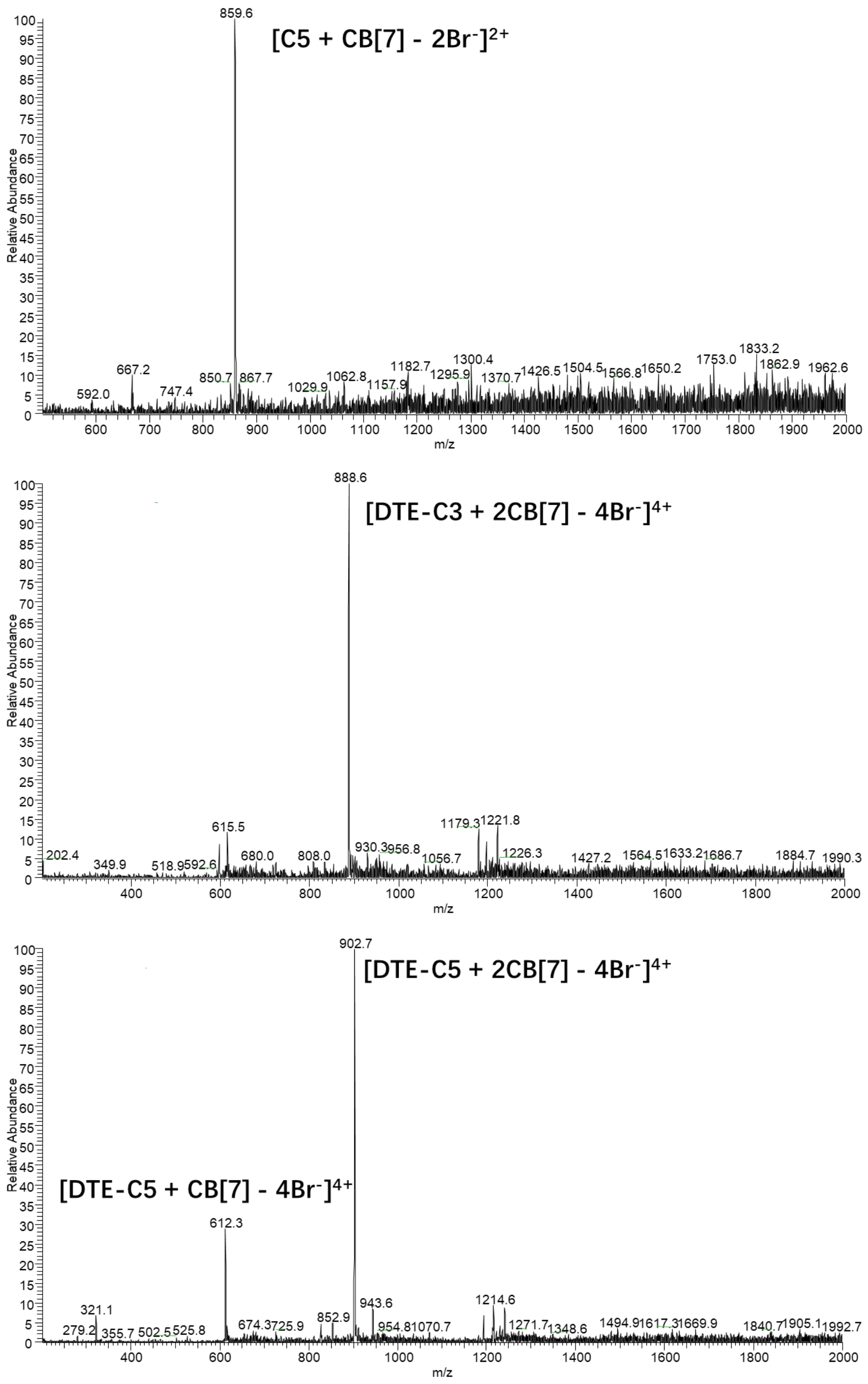

Figure S12. ESI-MS of C3⊂CB[7], C5⊂CB[7], DTE-C3⊂CB[7], and DTE-C5⊂CB[7].

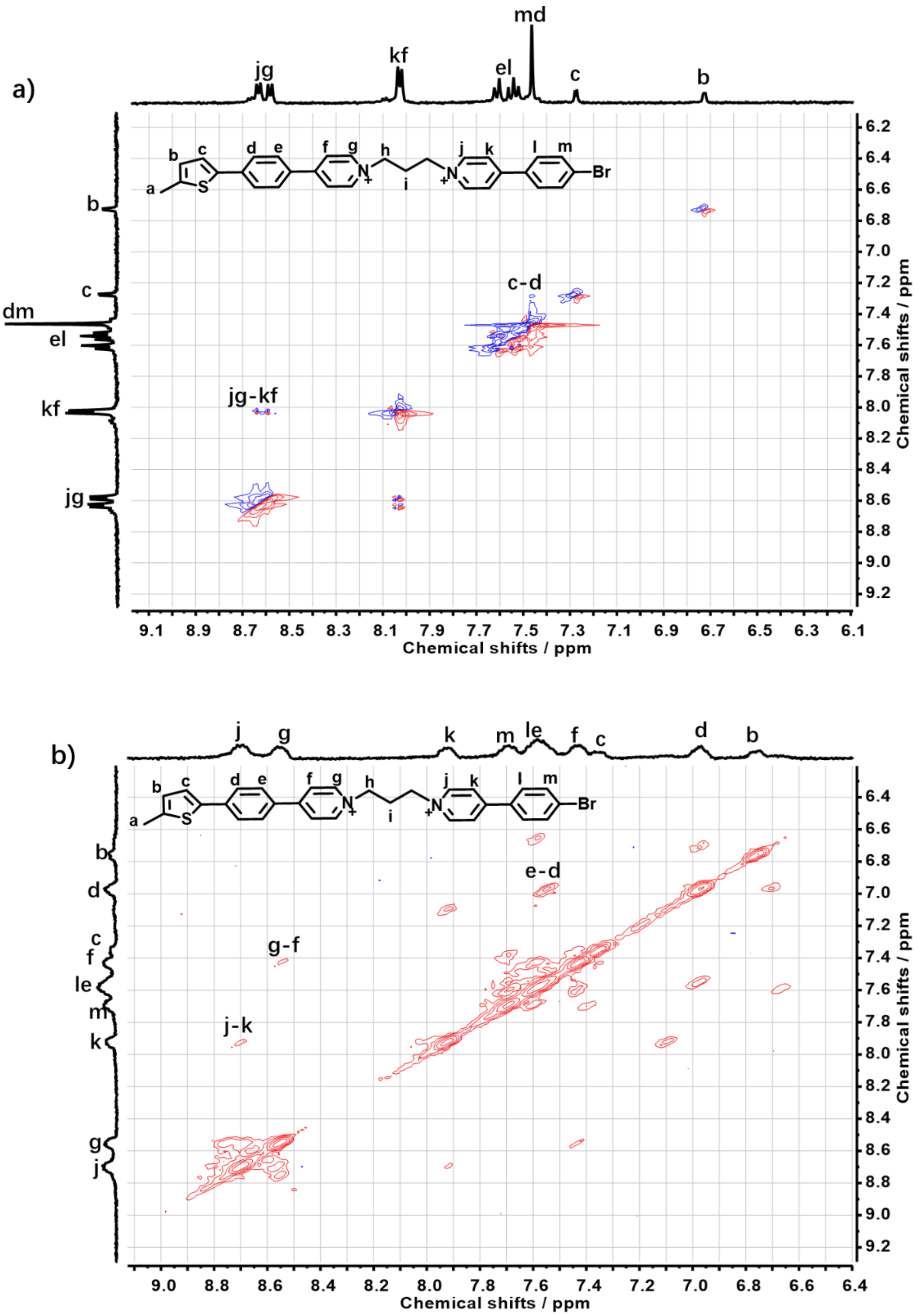

Figure S13. a) COSY of C3. b) 2D ^1H-^1H ROESY of C3⊂CB[7]. (D_2O/MeOD=5/1, v/v, 298 K)

2. Characterization of Luminous Properties

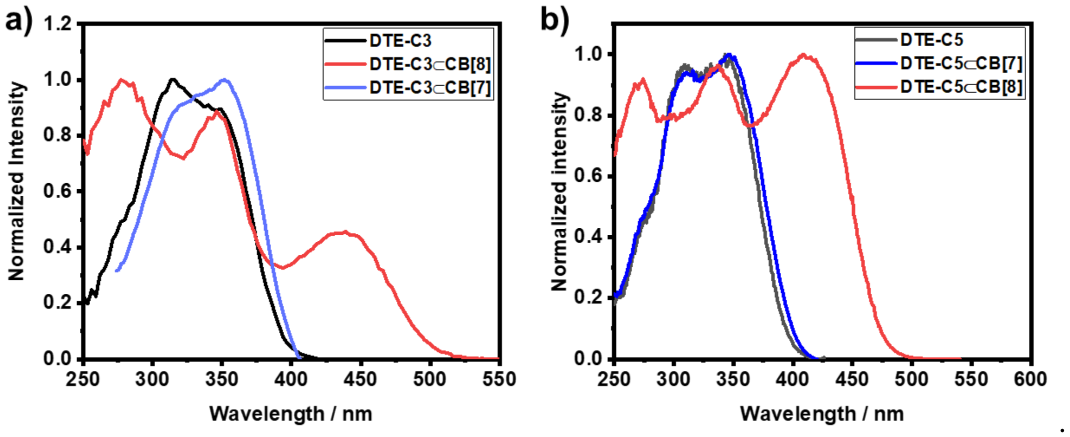

Figure S14. a) Normalized excitation spectra of DTE-C3, DTE-C3⊂CB[7] and DTE-C3⊂CB[8]. b) Normalized excitation spectra of DTE-C5, DTE-C5⊂CB[7] and DTE-C5⊂CB[8].

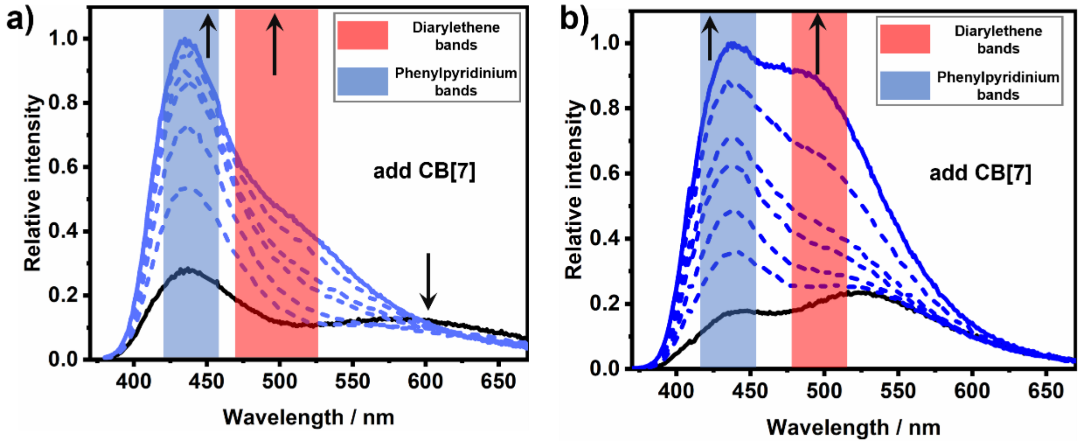

Figure S15. Photoluminescence spectral changes of a) DTE_{OF}-C3 and b) DTE_{OF}-C5 after adding 0.0-2.0 equivalent CB[7].

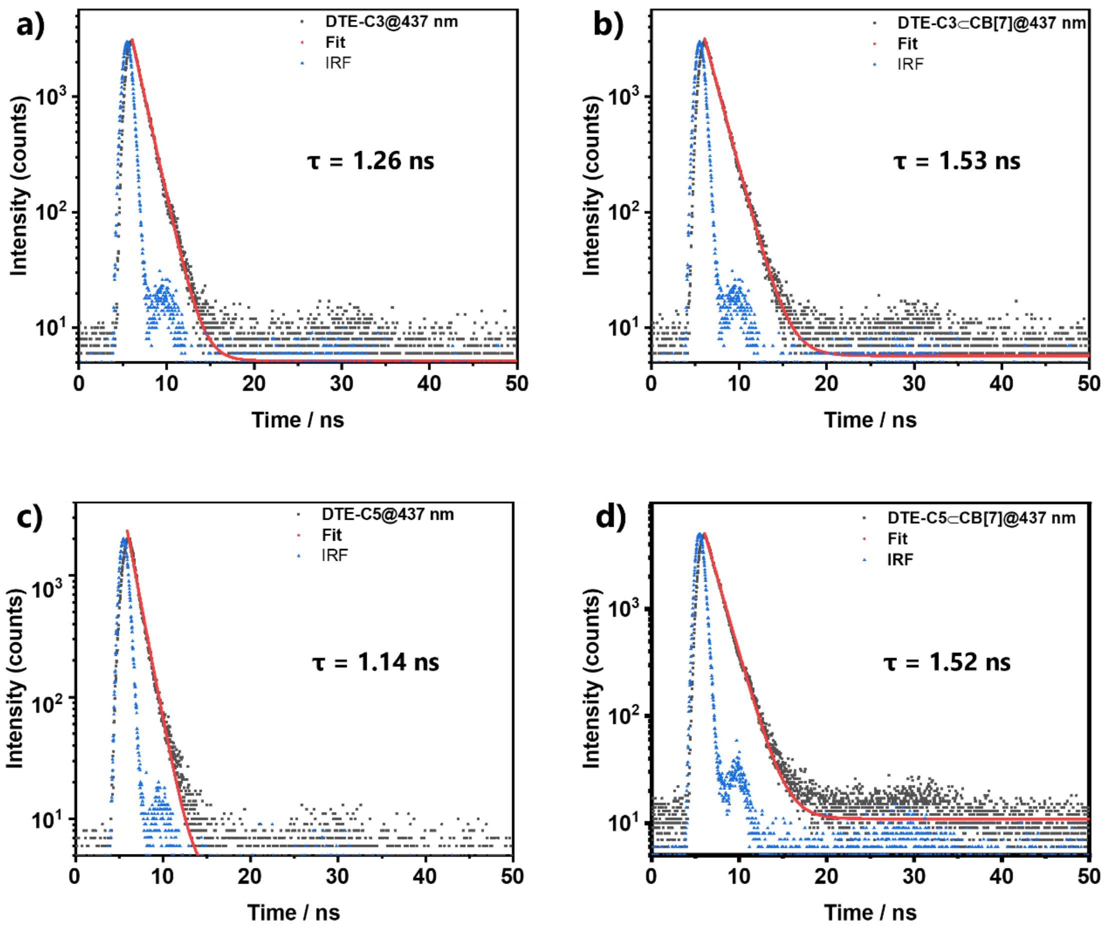

Figure S16. Time-resolved PL decay of a) DTE_{OF}-C3, b) DTE_{OF}-C3⊂CB[7], c) DTE_{OF}-C5, and d) DTE_{OF}-C5⊂CB[7] at room temperature.

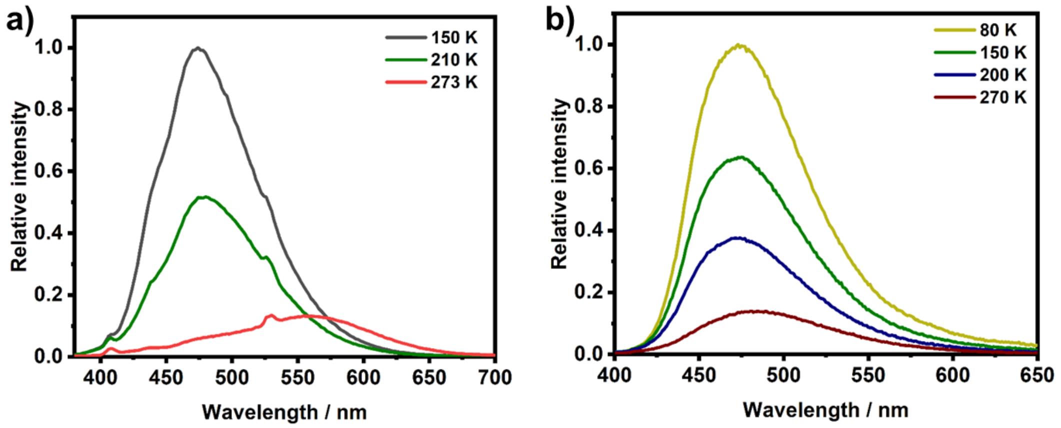

Figure S17. Varied temperature photoluminescence spectra of a) DTE_{OF}-C3⊂CB[8] and b) DTE_{OF}-C5⊂CB[8]. ([DTE_{OF}-C3] = [DTE_{OF}-C5] =8 μM, [CB[8]] = 16 μM)

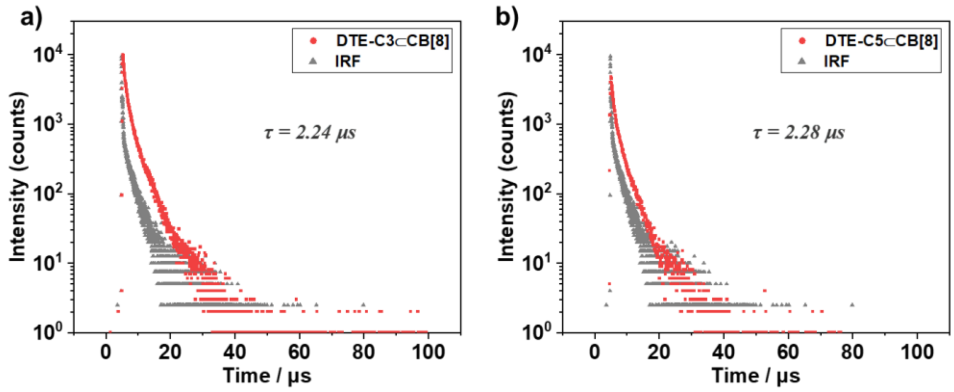

Figure S18. Time-resolved PL decay of a) DTE_{OF}-C3⊂CB[8] at 600 nm and b) DTE_{OF}-C5⊂CB[8] at 551 nm at room temperature.

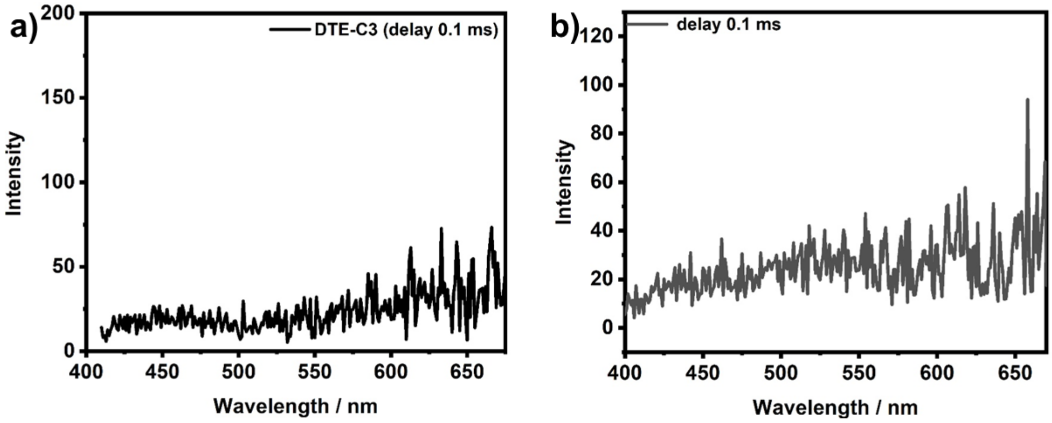

Figure S19. Phosphorescence spectra of a) DTE_{OF}-C3 and b) DTE_{OF}-C5. ([DTE_{OF}-C3] = [DTE_{OF}-C5] = 8 μM, delayed time of 0.1 ms)

3. Characterization of Photochromic Performance

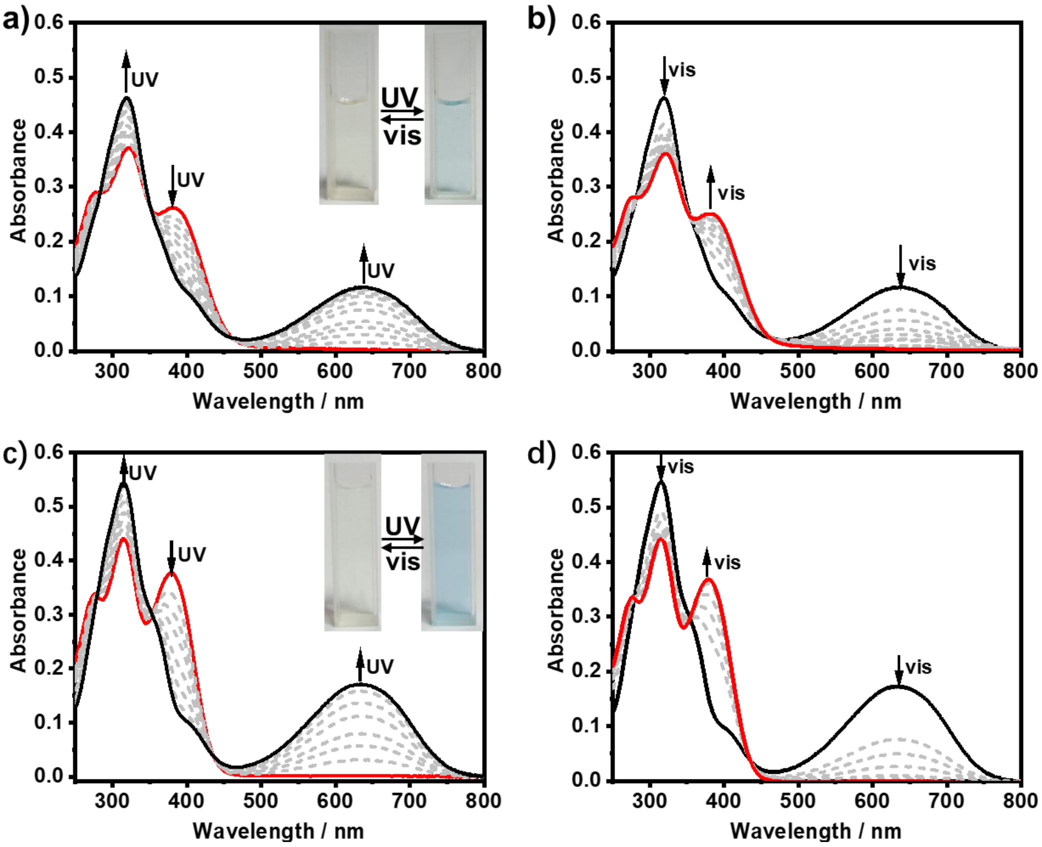

Figure S20. UV/vis spectral changes of DTE-C3 upon a) 365 nm and b) > 600 nm light irradiation. ([DTE-C3] = 6.7 µM, in H_2O, 298 K). UV/vis spectral changes of DTE-C5 upon c) 365 nm and d) > 600 nm light irradiation Inset: photographic images of DTE_{OF}-Cn and DTE_{CF}-Cn. ([DTE-C5] = 8.0 µM, in H_2O, 298 K).

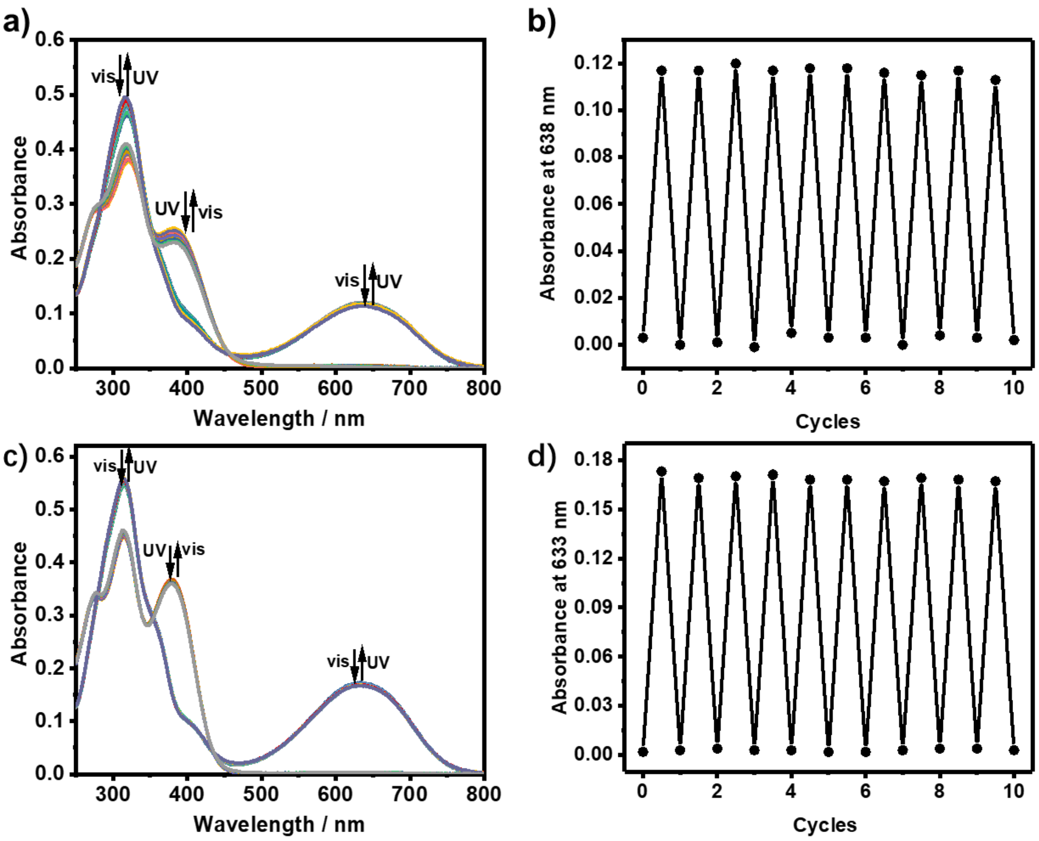

Figure S21. Fatigue resistance of DTE-C3 (a, b) and DTE-C5 (c, d) upon alternating UV and visible light irradiation ([DTE-C3] = 6.7 µM, [DTE-C5] = 8.0 µM, in H_2O).

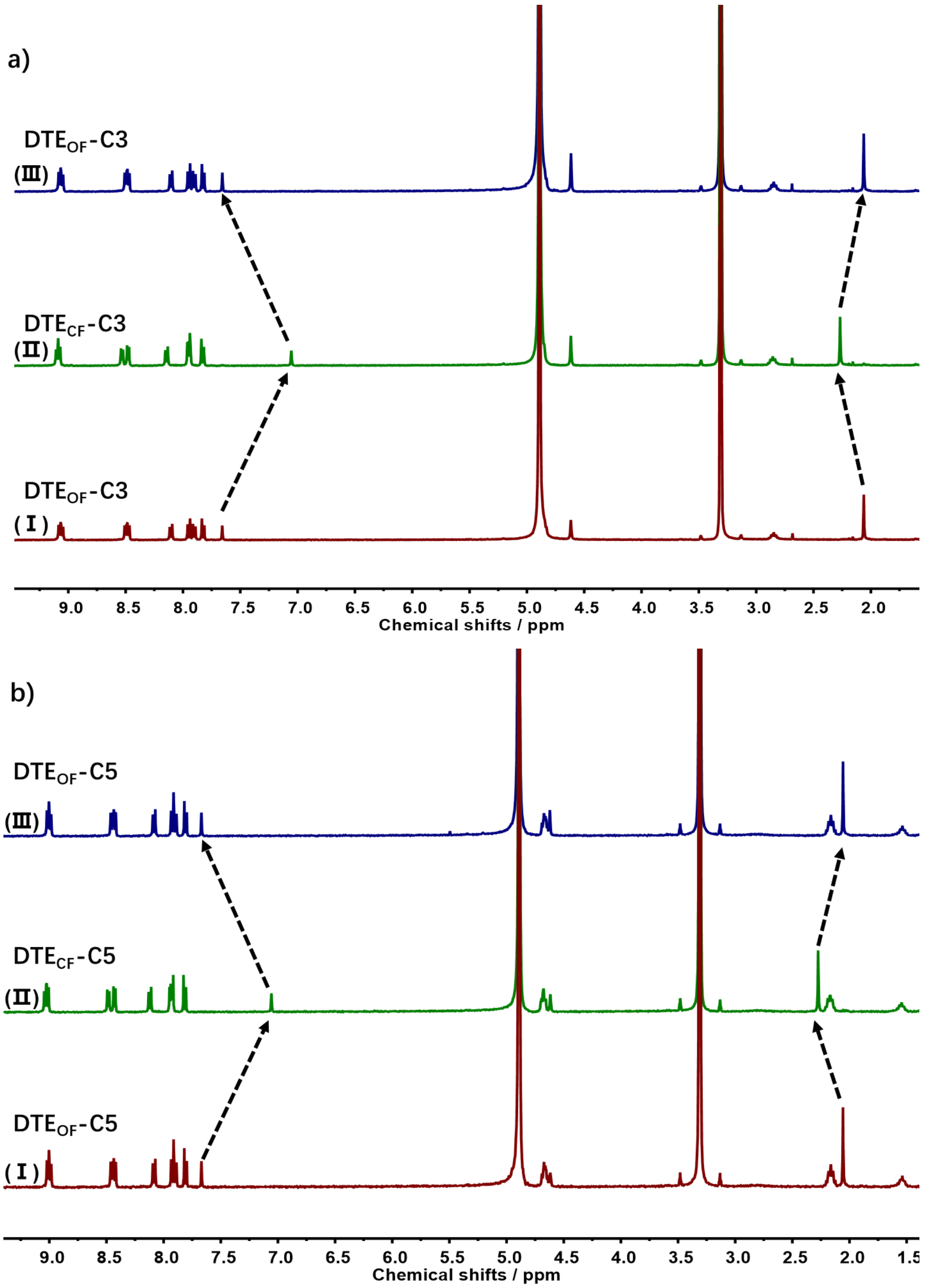

Figure S22. a) ^{1}H NMR spectral changes of (Ⅰ) DTE-C3 upon (Ⅱ) UV (λ = 365 nm) and (Ⅲ) visible (λ > 600 nm) light irradiation. b)^{1}H NMR spectral changes of (Ⅰ) DTE-C5 upon (Ⅱ) UV (λ = 365 nm) and (Ⅲ) visible (λ > 600 nm) light irradiation (400 MHz, MeOD, 298 K).

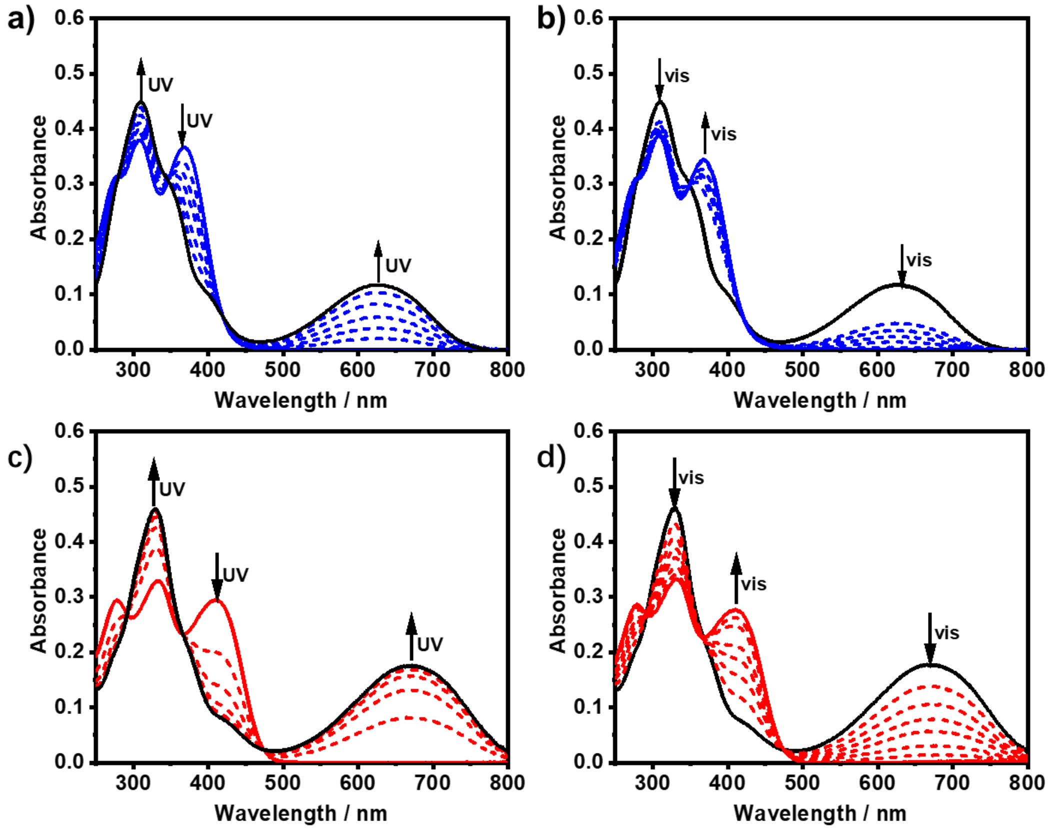

Figure S23. UV/vis spectral changes of DTE-C5⊂CB[7] upon a) 365 and b) > 600 nm light irradiation. UV/vis spectral changes of DTE-C5⊂CB[8] upon c) 365 and d) > 600 nm light irradiation ([DTE-C5] = 8.0 μM, [CB[7]] = 16.0 μM, [CB[8]] = 16.0 μM, in H_2O, 298 K).

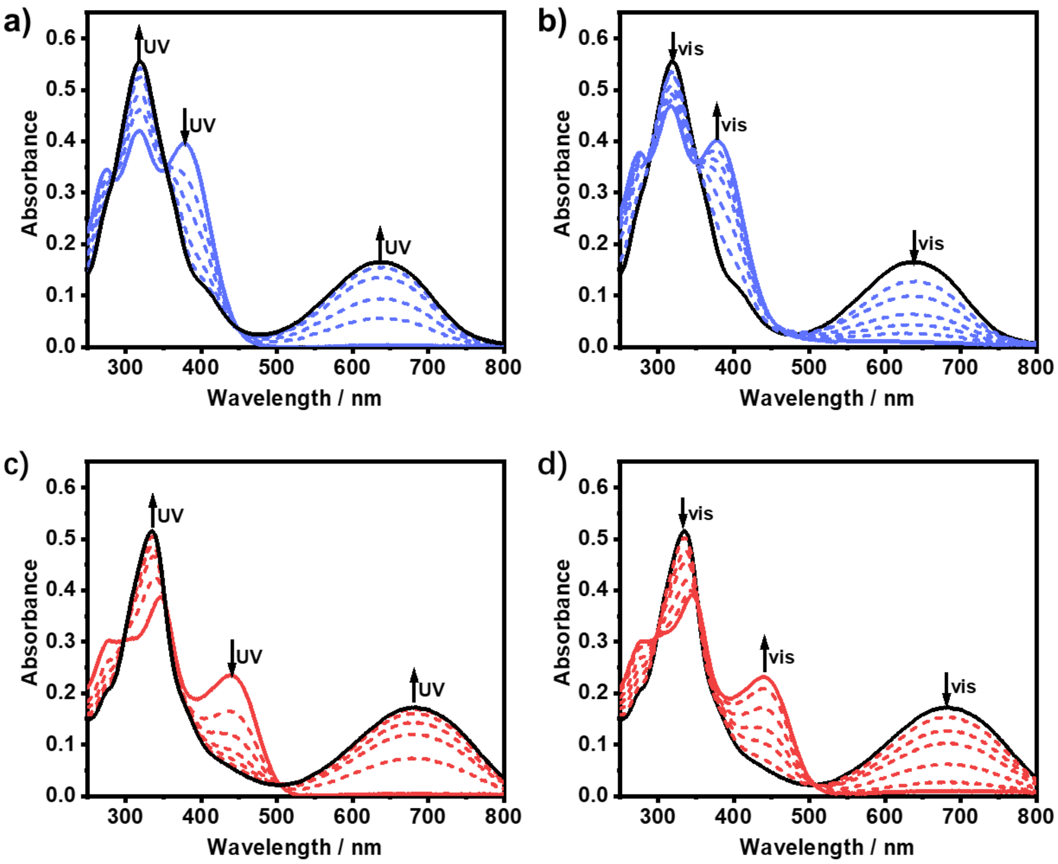

Figure S24. UV/vis spectral changes of DTE-C3⊂CB[7] upon a) 365 and b) > 600 nm light irradiation ([DTE-C3] = 8.0 μM, [CB[7]] = 16.0 μM, in 1%MeOH-H_2O, 298 K). UV/vis spectral changes of DTE-C3⊂CB[8] upon c) 365 and d) > 600 nm light irradiation ([DTE-C3] = 8.0 μM, [CB[8]] = 16.0 μM, in 1%MeOH-H_2O, 298 K).

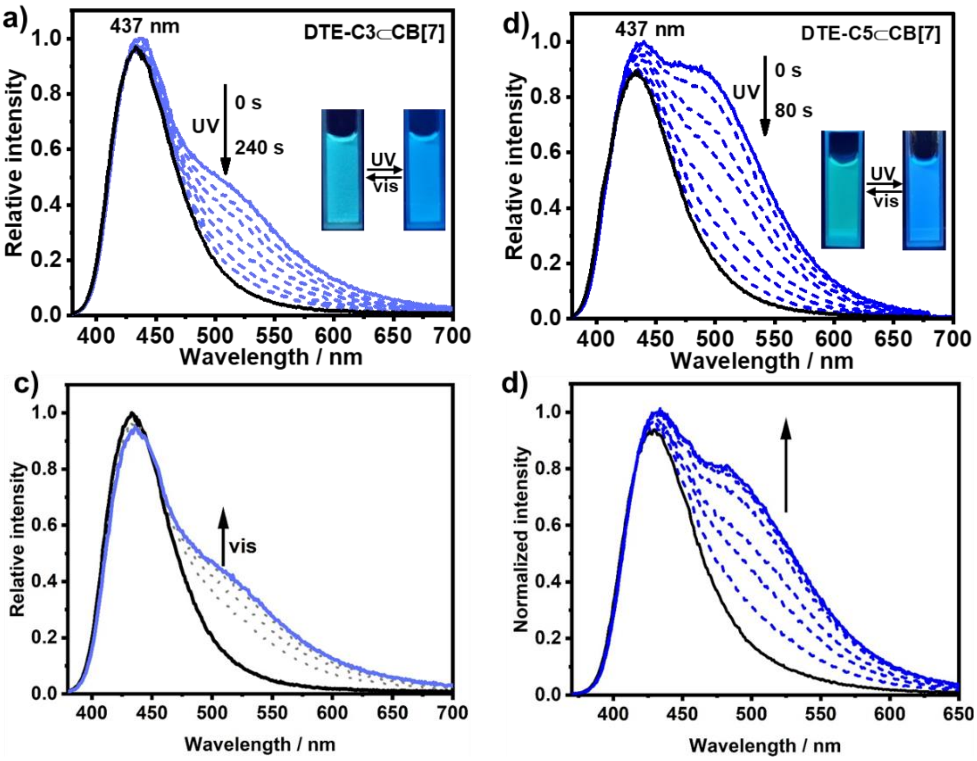

Figure S25. Photoluminescence spectral changes of DTE_{OF}-C3⊂CB[7] upon a)365 nm and c) > 600 nm light irradiation. ([DTE_{OF}-C3] = 8 μM, [CB[7]] = 16 μM, in 1%MeOH-H_2O). Photoluminescence spectral changes of DTE_{OF}-C5⊂CB[7] upon b) 365 nm and d) > 600 nm light irradiation. ([DTE_{OF}-C5] = 8 μM, [CB[7]] = 16 μM, in H_2O).

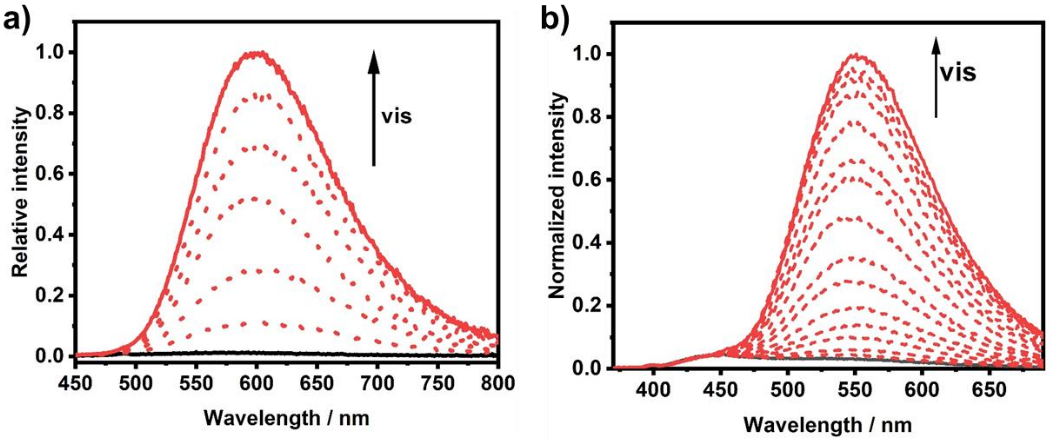

Figure S26. a) Photoluminescence spectral changes of DTE_{CF}-C3⊂CB[8] upon > 600 nm light irradiation. ([DTE_{CF}-C3] = 8 μM, [CB[8]] = 16 μM, in 1%MeOH-H_2O). b) Photoluminescence spectral changes of DTE_{CF}-C5⊂CB[8] upon > 600 nm light irradiation. ([DTE_{CF}-C5] = 8 μM, [CB[8]] = 16 μM, in H_2O)

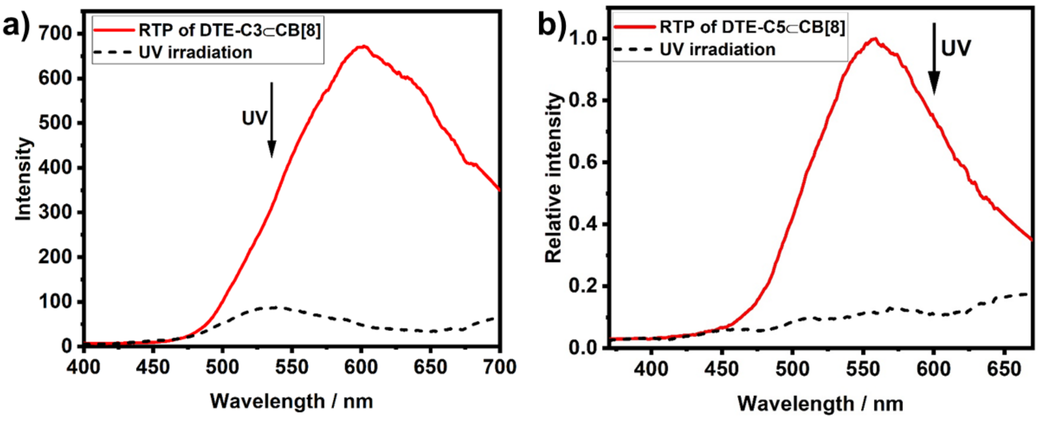

Figure S27. a) Phosphorescence spectral changes of DTE_{OF}-C3⊂CB[8] upon 365 nm light irradiation. b) Phosphorescence spectral changes of DTE_{OF}-C5⊂CB[8] upon 365 nm light irradiation. (delayed time of 0.1 ms.)

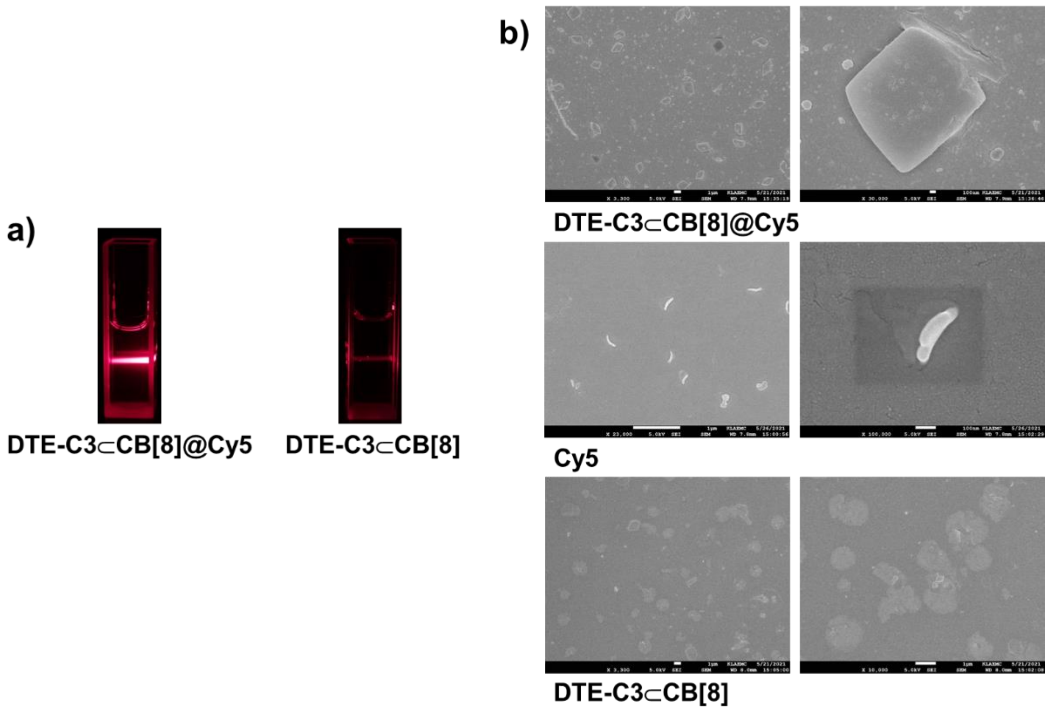

Figure S28. a) The Tyndall effect of DTE_{OF}-C3⊂CB[8] and DTE_{OF}-C3⊂CB[8]@Cy5. b) SEM image of DTE_{OF}-C3⊂CB[8] @Cy5, Cy5 and DTE_{OF}-C3⊂CB[8].

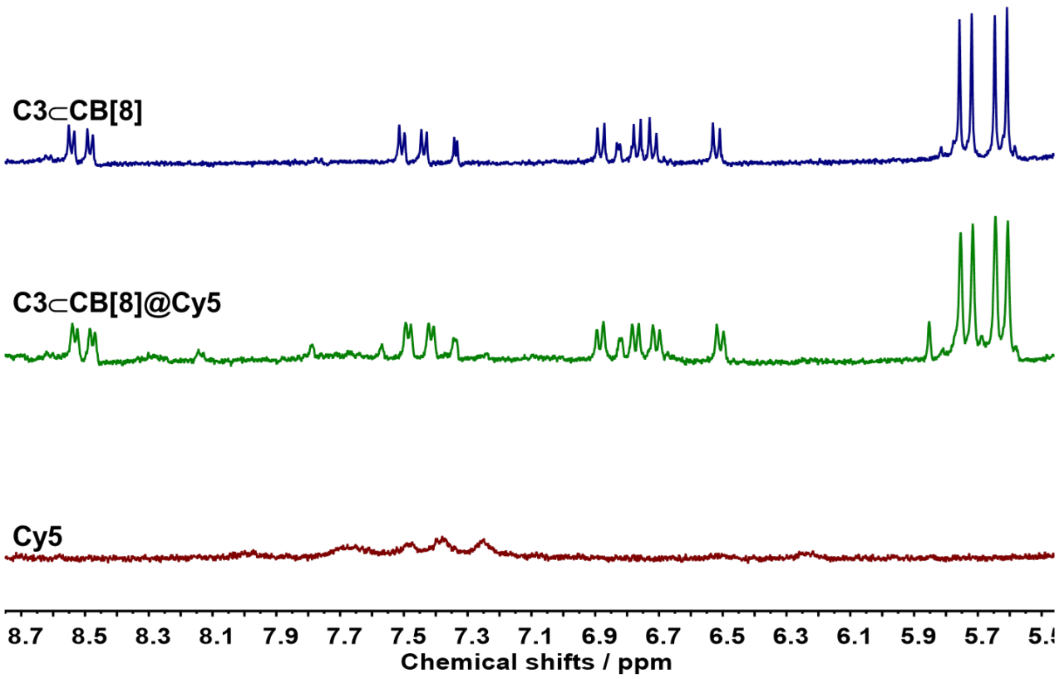

Figure S29. ^{1}H NMR spectra of C3⊂CB[8], C3⊂CB[8]@Cy5, and Cy5.

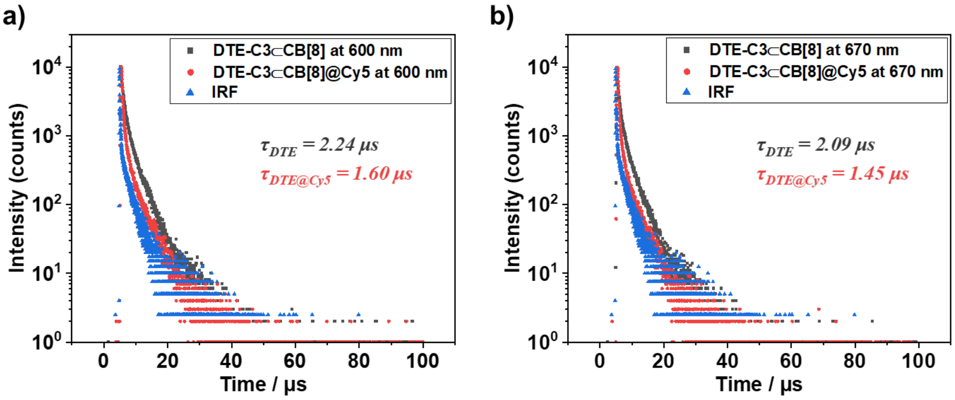

Figure S30. Time-resolved PL decay of DTE$_{OF}$-C3⊂CB[8] and DTE$_{OF}$-C3⊂CB[8]@Cy5 a) at 600 nm and b) at 670 nm.

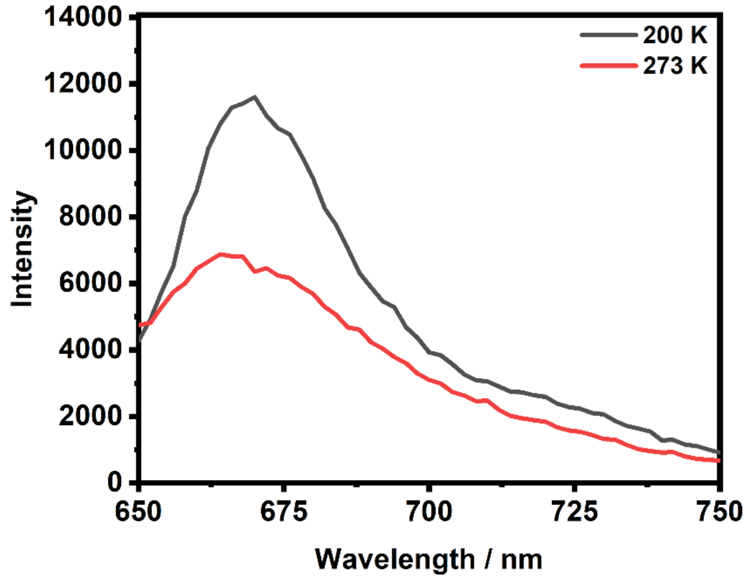

Figure S31. Varied temperature photoluminescence spectra of DTE_{OF}-C3⊂CB[8]@ Cy5.

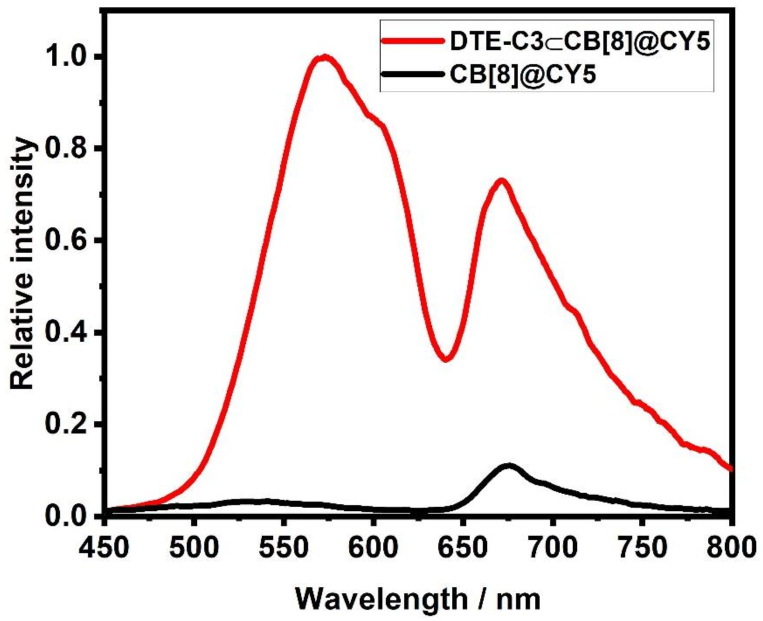

Figure S32. Photoluminescence spectrum of DTE_{OF}-C3⊂CB[8]@Cy5 and CB[8]@ Cy5.

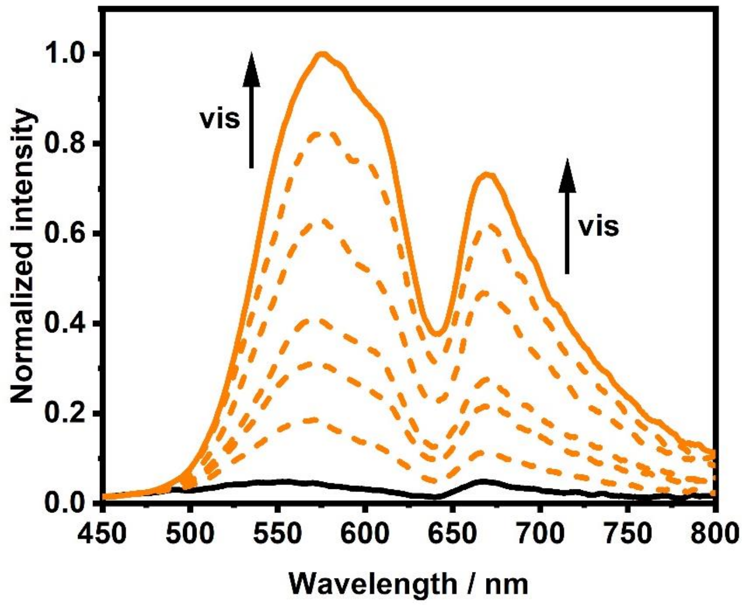

Figure S33. Photoluminescence spectrum changes of DTE_{CF}-C3⊂CB[8]@Cy5 upon irradiating with > 600 nm visible light.

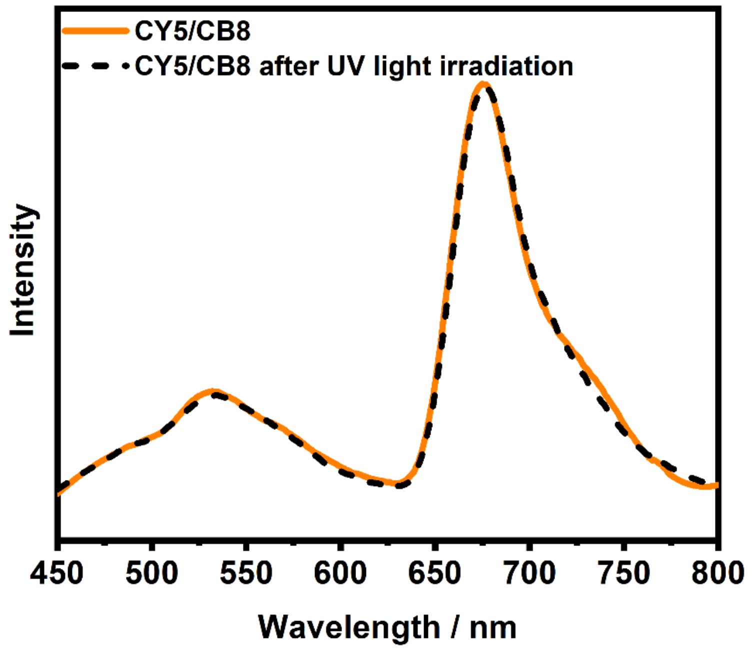

Figure S34. Photoluminescence spectrum change of CB[8]@Cy5 upon 365 nm light irradiation.

4. Measurement for Quantum Yield and Lifetime

Table S1. Photophysical data of DTE-Cn and assemblies.

Compound	λ_{ex}/nm	λ_{flu}/nm	λ_{pho}/nm	τ/ns	τ/μs	Φ_{flu}/%	Φ_{pho}/%	k_{ISC} /10^6	k_r^{Pho} /s^{-1}	k_{nr}^{Pho} /10^5 s^{-1}
DTE-C3	360	437<br>586	ND	1.26<br>0.99	ND	1.80	ND	NC	NC	NC
DTE-C3⊂CB[7]	360	437<br>510	ND	1.53<br>1.09	ND	5.49	ND	NC	NC	NC
DTE-C3⊂CB[8]	360	ND	551	ND	2.24	ND	1.71	NC	7600	4.38
DTE-C5	350	437<br>526	ND	1.14<br>1.42	ND	2.15	ND	NC	NC	NC
DTE-C5⊂CB[7]	350	437<br>485	ND	1.52<br>1.51	ND	5.70	ND	NC	NC	NC
DTE-C5⊂CB[8]	350	ND	600	ND	2.28	ND	2.43	NC	10657	4.28

The intersystem crossing rate constant, $k_{ISC}= \Phi_{Phos}/\tau_{Fluo}$.

The radiative decay rate constant of phosphorescence, $k_r^{Pho} = \Phi_{phos}/\tau_{Phos}$.

The nonradiative decay rate constant of phosphorescence, $k_{nr}^{Pho} = (1-\Phi_{Phos})/\tau_{Phos}$.

ND: not detected.

NC: not calculated.

5. Synthetic Routes and Characterization of Compound

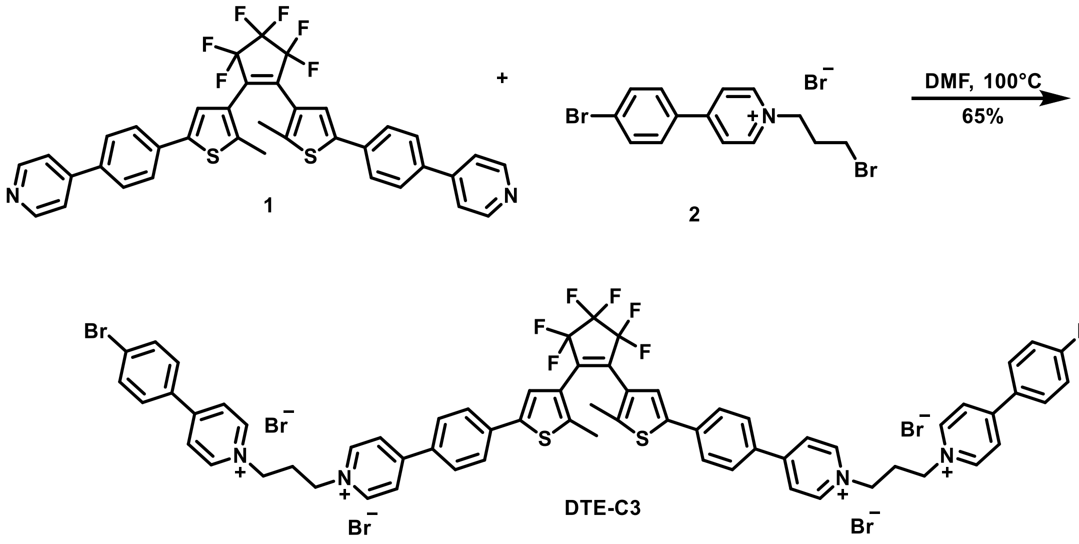

Scheme S1. Synthetic route of DTE-C3.

Compound 2 were synthesized by known procedure and the characterization data were in accordance with the reference.[1]

Synthesis of DTE-C3. Compound 1 (30 mg, 0.03 mmol) and 2 (75 mg, 0.17 mmol) were dissolved in dry DMF (0.5 ml). The reaction was stirred at 100 °C for 48 h. After cooled to room temperature, the resulting suspension was filtered and the solid washed repeatedly with dry DCM, and then dried in vacuo to obtain the yellow-green solid DTE-C3. Yield: 50 mg, 65%.

^{1}H NMR (400 MHz, CD_3OD-d_4, δ) 9.07 (d, J = 7.2 Hz, 4H, Ar H), 9.05 (d, J = 6.4 Hz, 4H, Ar H), 8.50 (d, J = 7.2 Hz, 4H, Ar H), 8.48 (d, J = 6.4 Hz, 4H, Ar H), 8.11 (d, J = 8.8 Hz, 4H, Ar H), 7.95 (d, J = 8.8 Hz, 4H, Ar H), 7.90 (d, J = 8.4 Hz, 4H, Ar H), 7.83 (d, J = 8.4 Hz, 4H, Ar H), 7.66 (s, 2H, Ar H), 4.84–4.79 (m, 8H, overlapped with residual H_2O, CH_2), 2.89–2.81 (m, 4H, CH_2), 2.06 (s, 6H, CH_3).

^{13}C NMR (100 MHz, CD_3OD-d_4, δ) 157.1, 157.1, 146.0, 145.8, 141.9, 139.6, 138.3, 134.0, 134.0, 133.9, 130.8, 130.0, 128.3, 127.4, 127.2, 126.1, 125.7, 125.4, 58.5, 58.3, 33.1, 14.5.

HRMS (ESI) *m/z*: $[M – 4Br^{-}]^{4+}$ calcd for $C_{65}H_{52}Br_6F_6N_4S_2$, 306.5465; found: 306.5472.

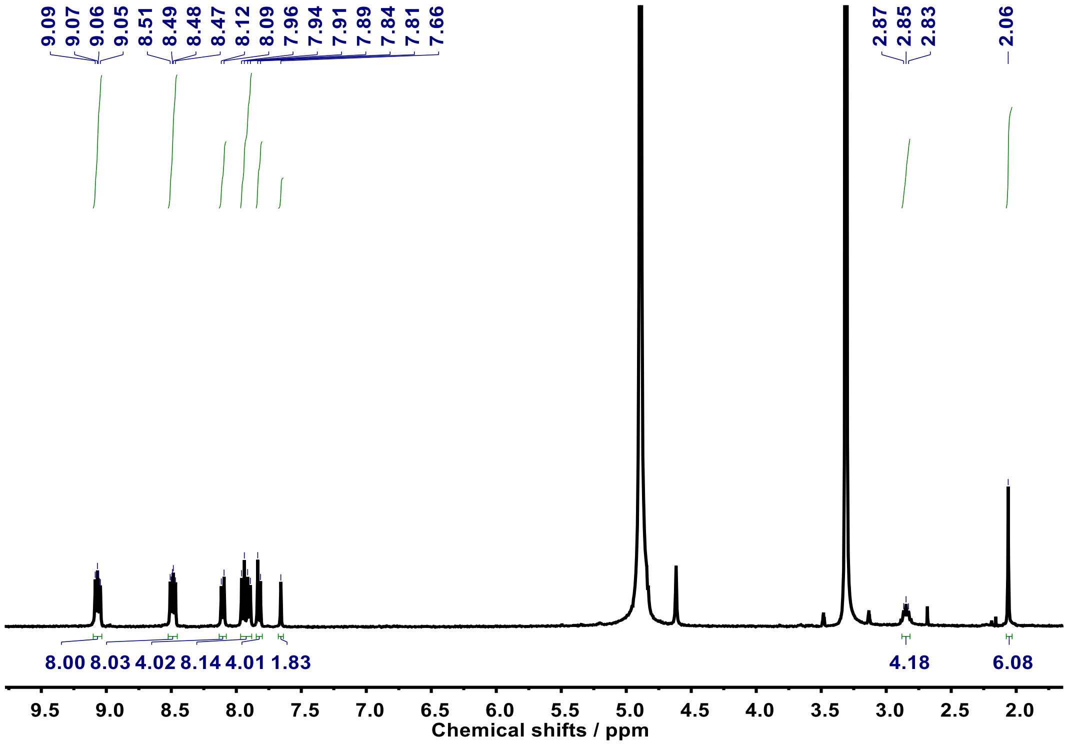

Figure S35. ^{1}H NMR spectrum (400 MHz, CD_3OD, 25 °C) of DTE-C3.

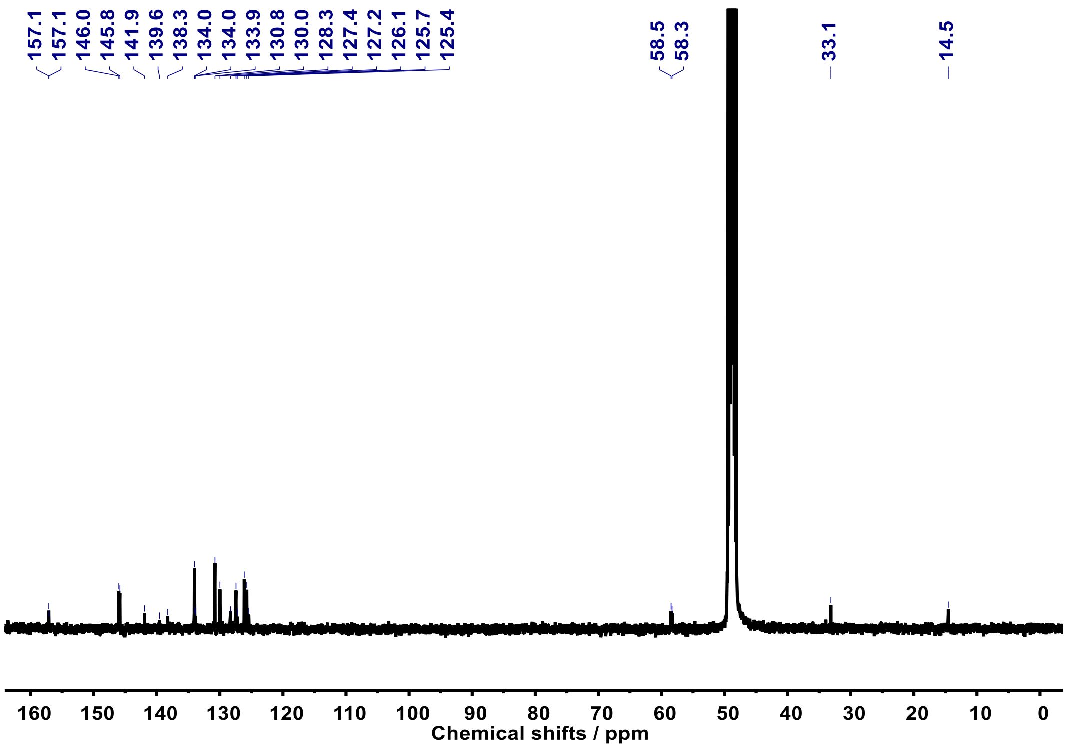

Figure S36. ^{13}C NMR spectrum (100 MHz, CD_3OD, 25 °C) of DTE-C3.

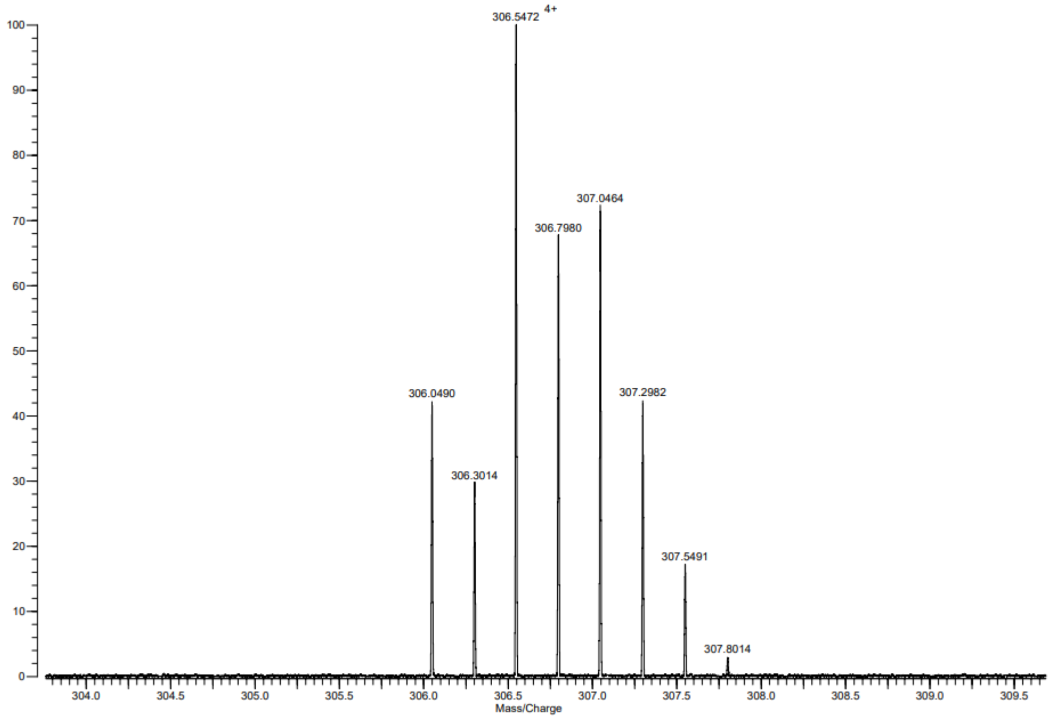

Figure S37. HR-MS of DTE-C3.

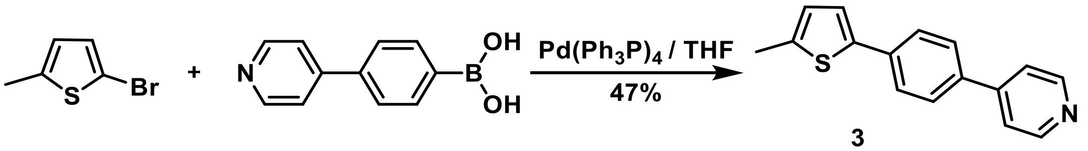

Scheme S2. Synthetic route of 3.

Synthesis of 3. 2-bromo-5-methyl thiophene (0.3 g, 1.7 mmol), (4-(pyridine-4-yl)phenyl)boronic acid (0.4 g, 2.0 mmol), and tetrakis(triphenylphosphine) palladium(0) (0.8 g, 0.69 mmol) were dissolved in a mixed solvent of 1,2-dimethoxy-ethan (10 ml) and water (2.5 ml). The mixture was stirred for 48 h at 90 °C. The resulting solution was poured into ice-cold water, extracted with ethyl acetate, and dried over $MgSO_4$. The solvent was evaporated under reduced pressure and the residue was subjected to silica gel column chromatography using a mixture of MeOH / dichloromethane as an eluent, affording a light gray solid substance. Yield: 0.2 g, 47%.

^{1}H NMR (400 MHz, $CDCl_3$-d_3, δ) 8.66 (d, 1H, J = 1.6 Hz, Ar H), 8.65 (d, 1H, J = 2.0 Hz, Ar H),7.65 (d, 4H, J = 2.4 Hz, Ar H), 7.53 (d, 1H, J = 1.6 Hz, Ar H), 7.52 (d, 1H, J = 1.6 Hz, Ar H), 7.19 (d, 2H, J = 4.0 Hz, Ar H), 7.19 (m, 1H, Ar H), 2.53 (s, 3H, CH_3).

^{13}C NMR (100 MHz, $CDCl_3$-d_3, δ) 150.3, 147.6, 140.9, 140.4, 136.3, 135.5, 127.4, 126.4, 126.0, 123.6, 121.2, 15.5.

HRMS (FTMALDI) m/z: $[M + H]^+$ calcd for $C_{16}H_{13}NS$, 252.0769; found: 252.0845.

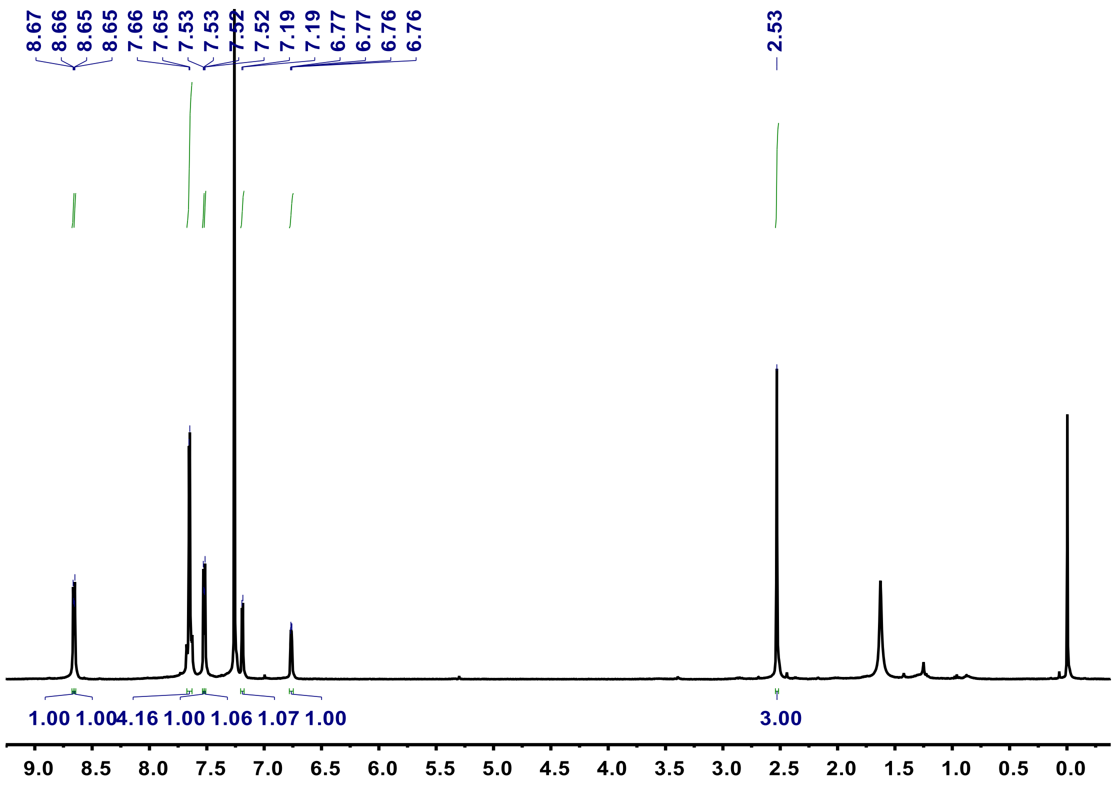

Figure S38. ^{1}H NMR spectrum (400 MHz, $CDCl_3$) of 3.

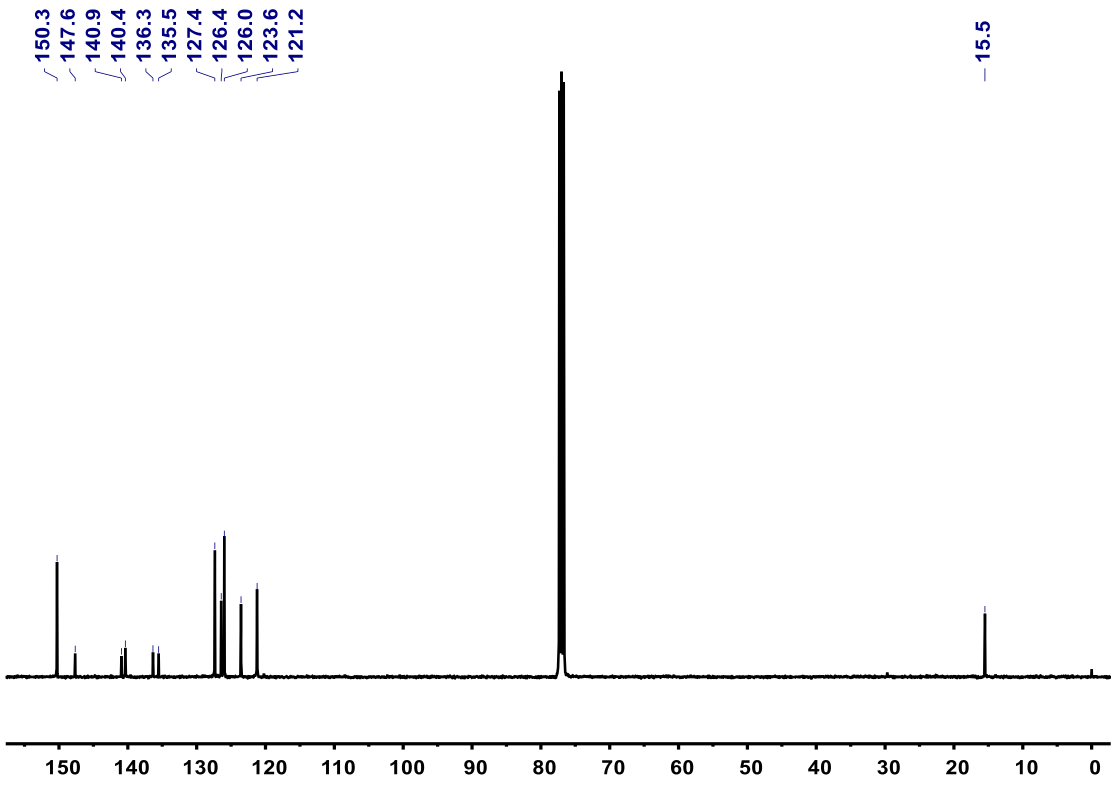

Figure S39. ^{13}C NMR spectrum (100 MHz, $CDCl_3$) of 3.

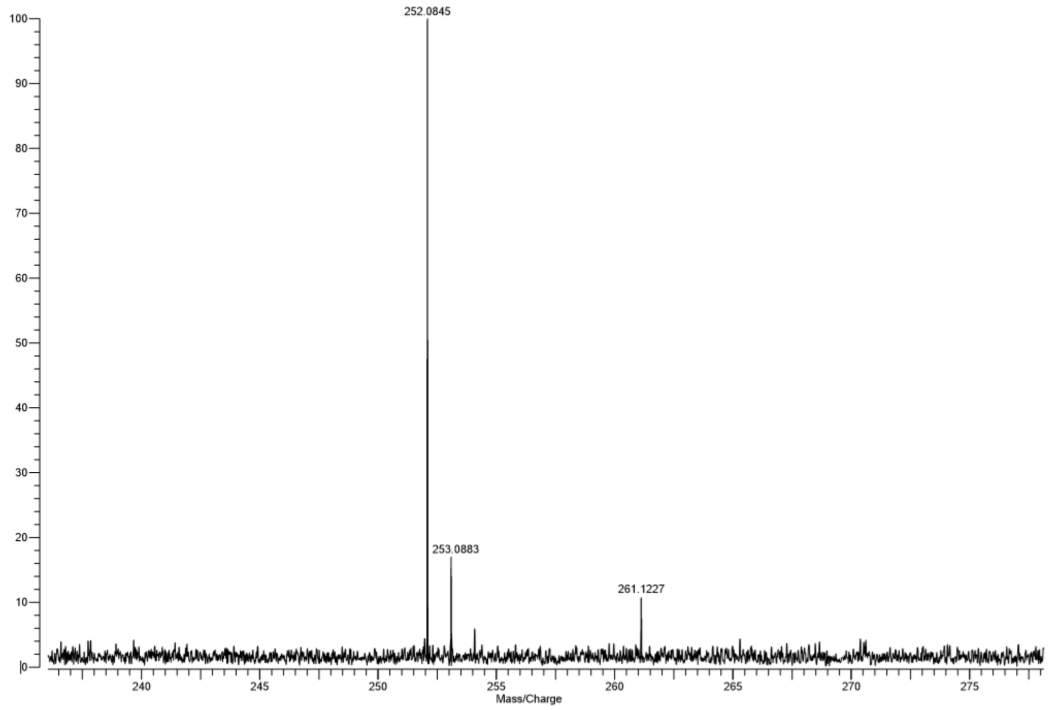

Figure S40. HR-MS of 3.

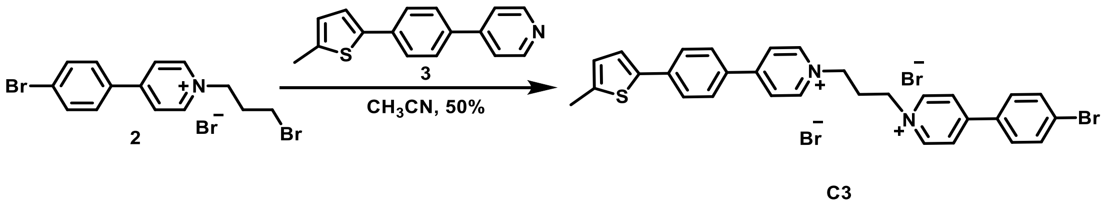

Scheme S3. Synthetic route of C3.

Synthesis of C3. Compound 3 (40 mg, 0.16 mmol) and 2 (76 mg, 0.17 mmol) were dissolved in dry CH_3CN (5 ml). The reaction was stirred at 95 °C for 3 d. After cooled to room temperature, the resulting suspension was filtered and the solid washed repeatedly with dry CH_3CN, and then dried in vacuo to obtain the yellow-green solid C3. Yield: 55 mg, 50%.

^1H NMR (400 MHz, CD_3OD-d_4, δ) 9.17 (d, J = 6.0 Hz, 2H, Ar H), 9.12 (d, J = 6.8 Hz, 2H, Ar H), 8.60 (d, J = 6.8 Hz, 4H, Ar H), 8.15 (d, J = 8.4 Hz, 2H, Ar H), 8.06 (d, J = 8.8 Hz, 2H, Ar H), 7.88 (d, J = 8.8 Hz, 2H, Ar H), 7.85 (d, J = 8.8 Hz, 2H, Ar H), 7.59 (d, J = 3.6 Hz, 1H, Ar H), 6.92 (dd, J = 1.2, 3.6 Hz, 1H, Ar H), 4.75 (q, J = 6.8 Hz, 4H, CH_2), 2.75–2.68 (m, 2H, CH_2), 2.51 (s, 3H, overlapped with residual DMSO, CH_3).

^{13}C NMR (100 MHz, CD_3OD-d_4, δ) 154.1, 153.8, 145.2, 145.0, 141.5, 139.3, 137.7, 132.8, 132.7, 131.5, 130.2, 129.1, 127.5, 126.5, 125.9, 125.8, 124.6, 124.0, 56.9, 56.7, 31.5, 15.3.

HRMS (ESI) m/z: $[M – 2Br^-]^{2+}$ calcd for $C_{30}H_{27}Br_3N_2S$, 263.0534; found: 263.0537.

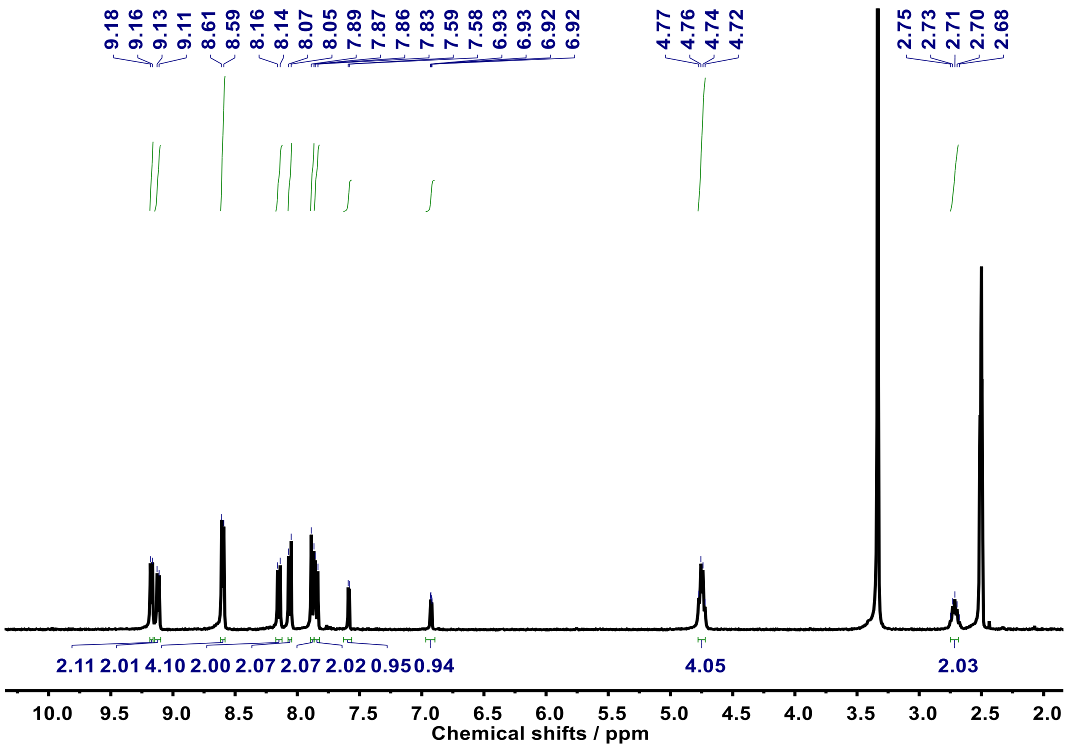

Figure S41. ^{1}H NMR spectrum (400 MHz, DMSO, 25 °C) of C3.

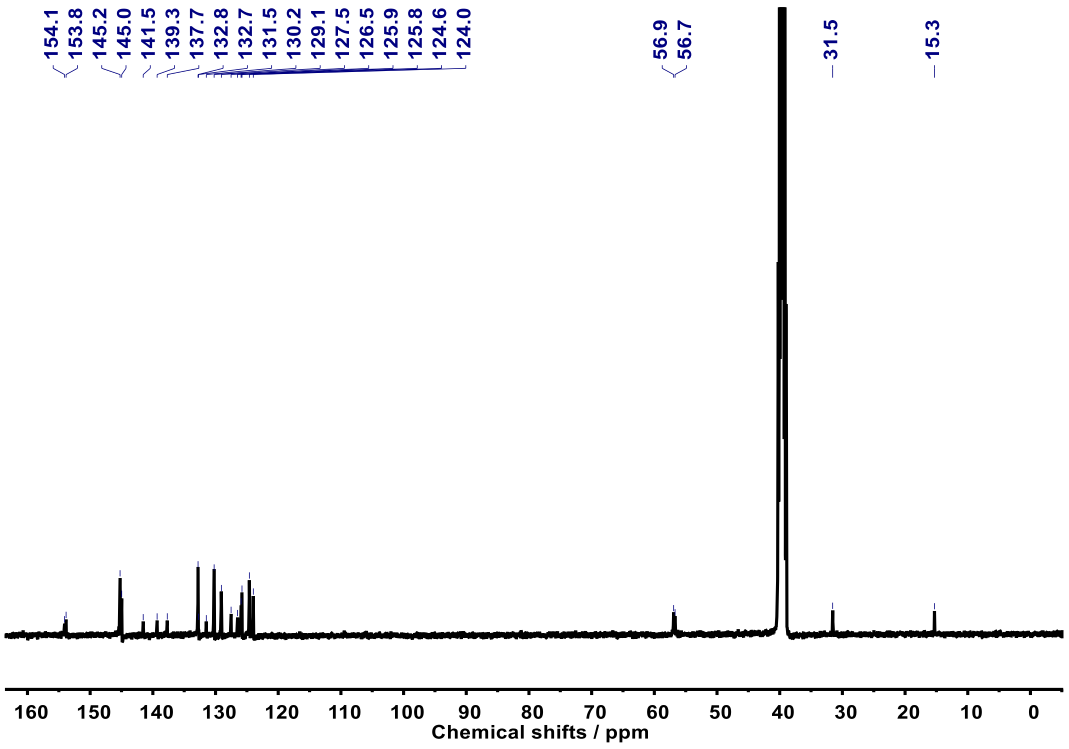

Figure S42. ^{13}C NMR spectrum (100 MHz, DMSO, 25 °C) of C3.

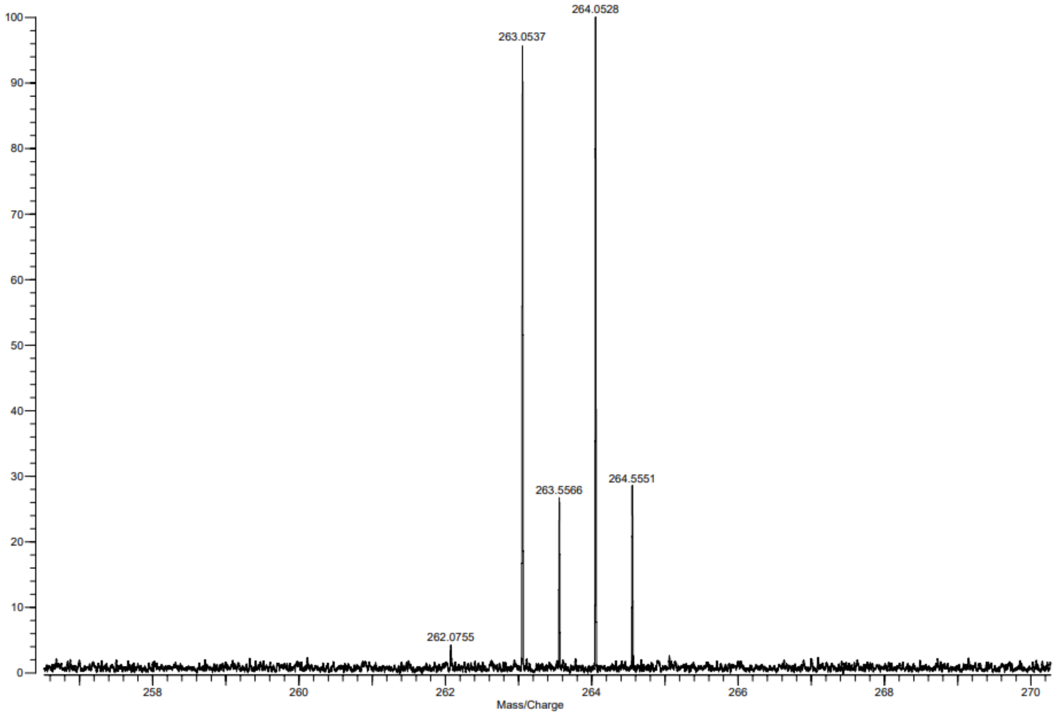

Figure S43. HR-MS of C3.

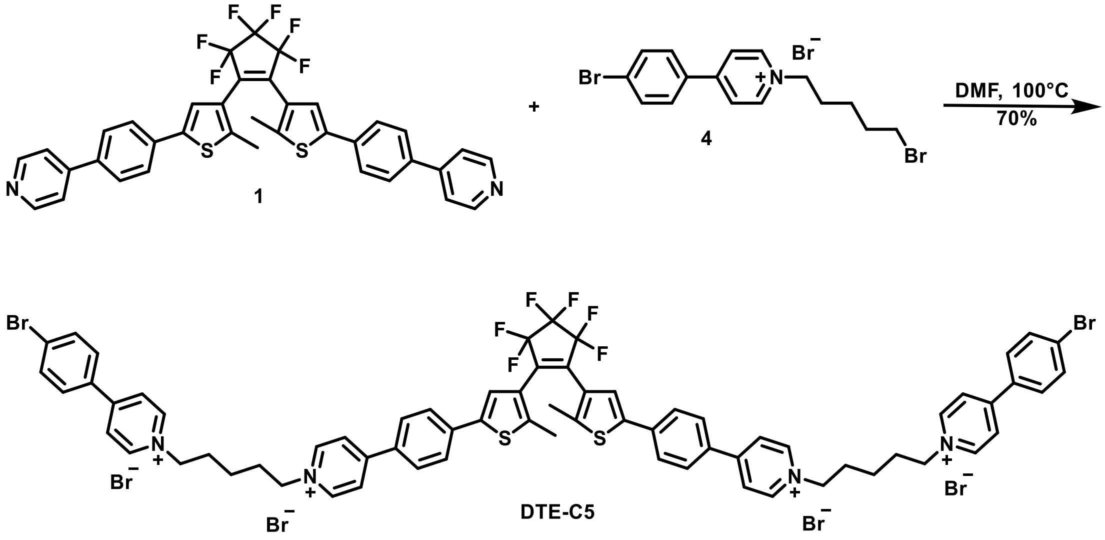

Scheme S4. Synthetic route of DTE-C5.

Compound 4 was synthesized by known procedure and the characterization data were in accordance with the reference.[1]

Synthesis of DTE-C5. Compound 1 (30 mg, 0.03 mmol) and 4 (75 mg, 0.16 mmol) were dissolved in dry DMF (0.5 ml). The reaction was stirred at 100 °C for 48 h. After cooled to room temperature, the resulting suspension was filtered and the solid washed repeatedly with dry DCM, and then dried in vacuo to obtain the yellow-green solid DTE-C5. Yield: 50 mg, 70%.

^{1}H NMR (400 MHz, CD_3OD-d_4, δ): 9.00 (t, J = 7.2 Hz, 8H, Ar H), 8.45 (d, J = 7.2 Hz, 4H, Ar H), 8.43 (d, J = 6.8 Hz, 4H, Ar H), 8.08 (d, J = 8.4 Hz, 4H, Ar H), 7.91 (t, J = 9.2 Hz, 8H, Ar H), 7.81 (d, J = 8.4 Hz, 4H, Ar H), 7.67 (s, 2H, Ar H), 4.69–4.64 (m, 8H, CH_2), 2.16 (t, J = 8.0 Hz, 8H, CH_2), 2.06 (s, 6H , CH_3), 1.56–1.51 (m, 4H, CH_2).

^{13}C NMR (100 MHz, CD_3OD-d_4, δ): 156.6, 156.6, 145.9, 145.7, 144.6, 142.0, 138.1, 134.0,

134.0, 130.7, 129.9, 128.1, 127.4, 127.2, 125.9, 125.5, 125.4, 61.5, 61.4, 31.3, 23.4, 14.6.

HRMS (ESI) *m*/*z*: [M – 4Br$^-$]$^{4+}$ calcd for $C_{69}H_{60}Br_6F_6N_4S_2$, 320.5622; found: 320.5627.

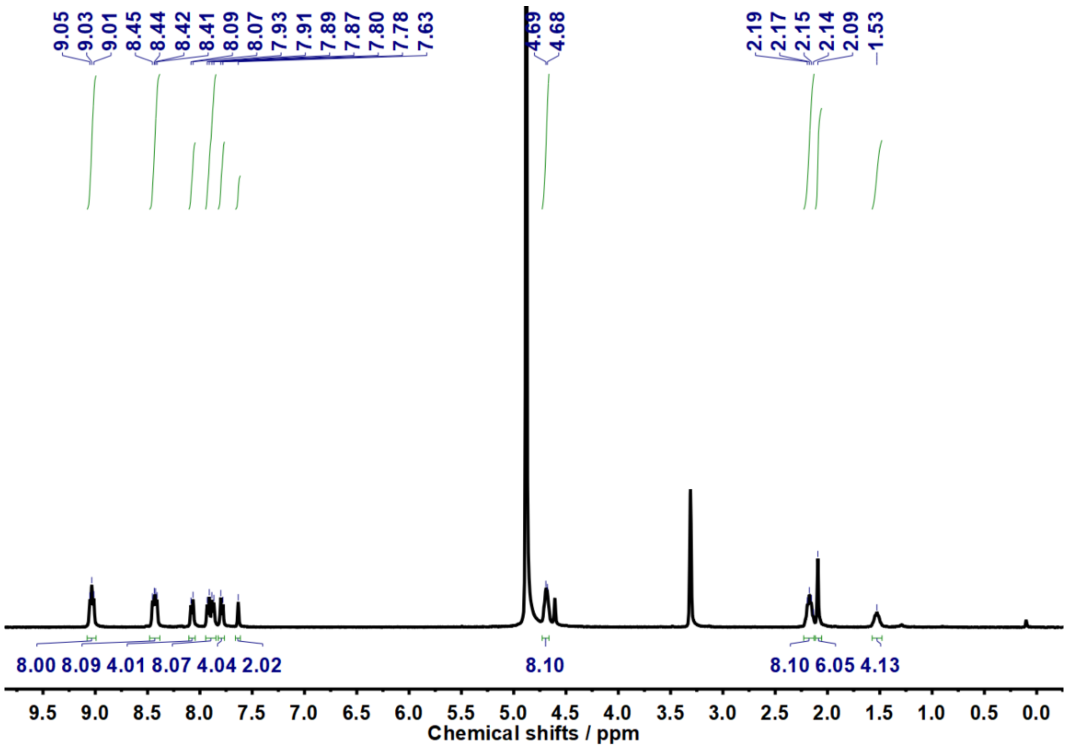

Figure S44. ^{1}H NMR spectrum (400 MHz, CD_3OD, 25 °C) of DTE-C5.

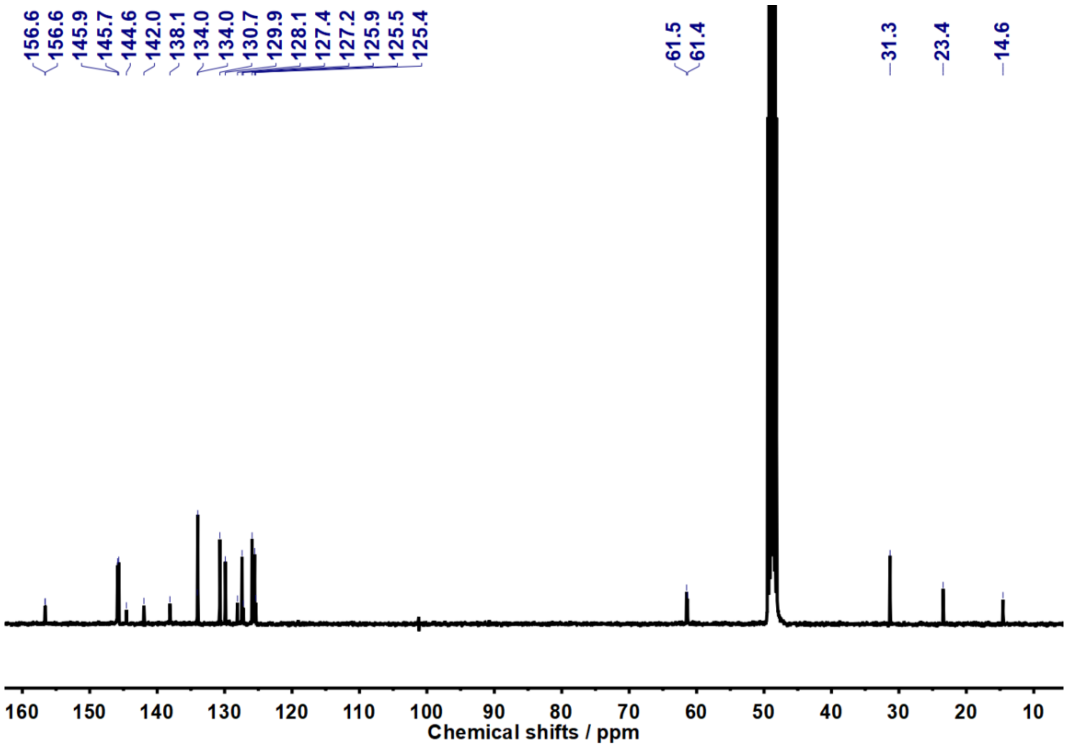

Figure S45. ^{13}C NMR spectrum (100 MHz, CD_3OD, 25 °C) of DTE-C5.

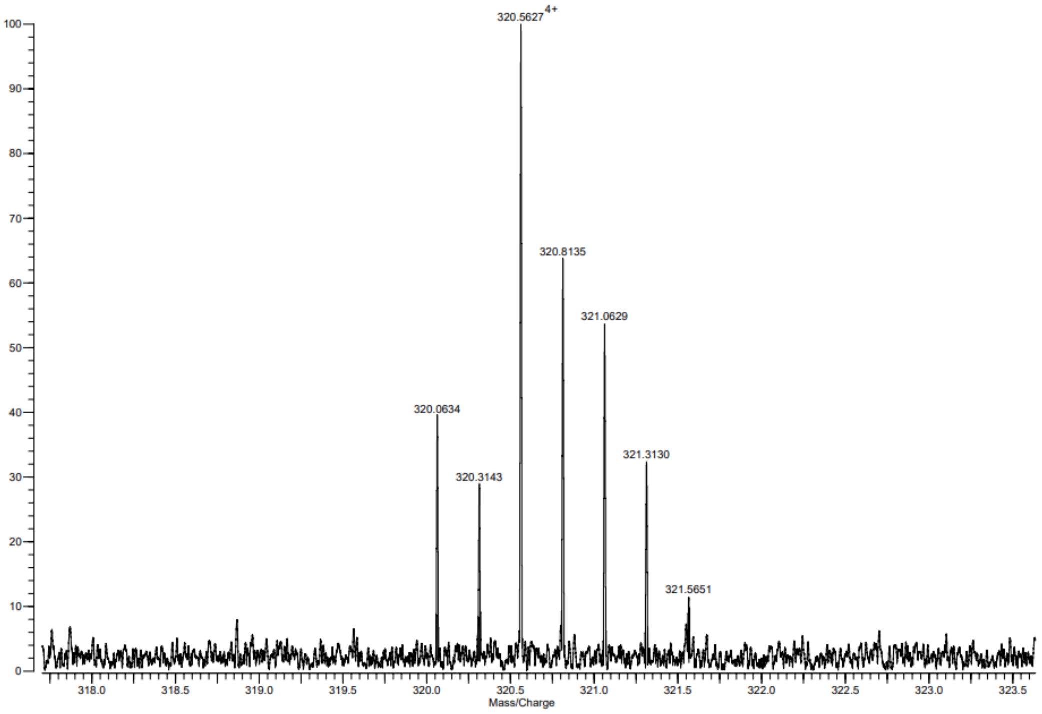

Figure S46. HR-MS of DTE-C5.

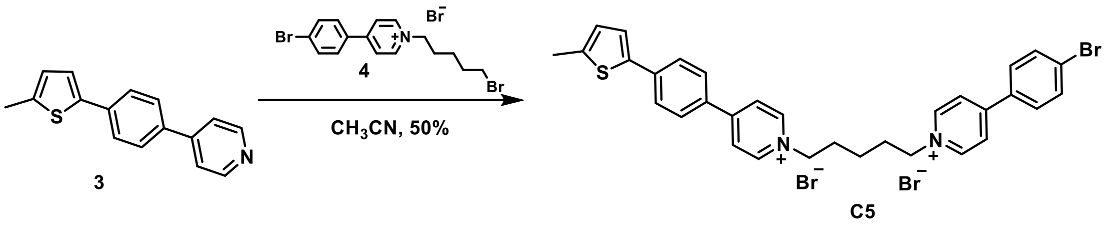

Scheme S5. Synthetic route of C5.

Synthesis of C5. Compound 3 (40 mg, 0.16 mmol) and 2 (80 mg, 0.17 mmol) were dissolved in dry CH_3CN (5 ml). The reaction was stirred at 95 °C for 3 d. After cooled to room temperature, the resulting suspension was filtered and the solid washed repeatedly with dry CH_3CN, and then dried in vacuo to obtain the yellow-green solid C3. Yield: 57 mg, 50%.

^{1}H NMR (400 MHz, DMSO-d_6, δ): 9.17 (d, J = 6.8 Hz, 2H, Ar H), 9.12 (d, J = 6.4 Hz, 2H, Ar

H), 8.56 (d, J = 6.4 Hz, 4H, Ar H), 8.13 (d, J = 8.4 Hz, 2H, Ar H), 8.04 (d, J = 8.4 Hz, 2H, Ar H), 7.87-7.82 (m, 4H, Ar H), 7.58 (d, J = 3.6 Hz, 1H, Ar H), 6.92 (d, J = 3.6 Hz, 1H, Ar H), 4.64-4.59 (m, 4H), 2.50 (s, 3H, overlapped with DMSO, CH_3), 2.04-2.00 (m, 4H, CH_2), 1.35-1.28 (m, 2H, CH_2).

^{13}C NMR (100 MHz, DMSO-d_6, δ): 153.8, 153.6, 145.0, 144.8, 141.5, 139.3, 137.6, 132.7, 131.5, 130.2, 129.0, 127.5, 126.4, 125.9, 125.7, 124.6, 123.9, 59.6, 59.3, 29.9, 21.9, 15.3.

HRMS (ESI) m/z: $[M - 2Br^-]^{2+}$, calcd for $C_{32}H_{31}Br_3N_2S$, 277.0690; found: 277.0693.

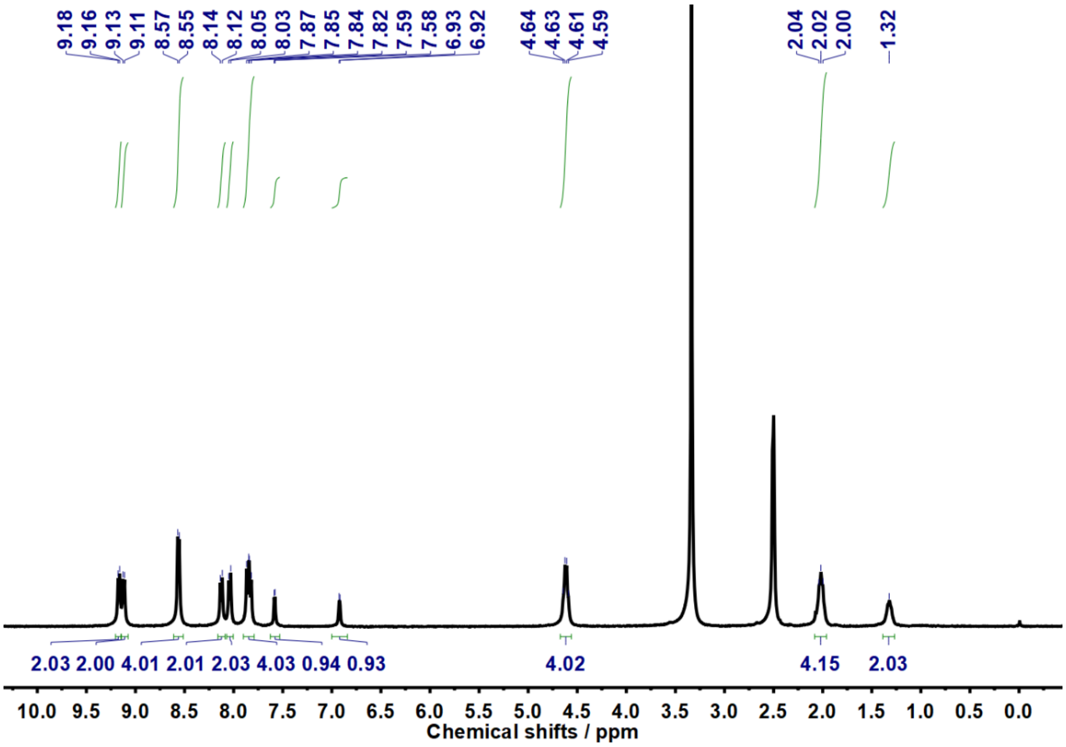

Figure S47. ^{1}H NMR spectrum (400 MHz, DMSO, 25 °C) of C5.

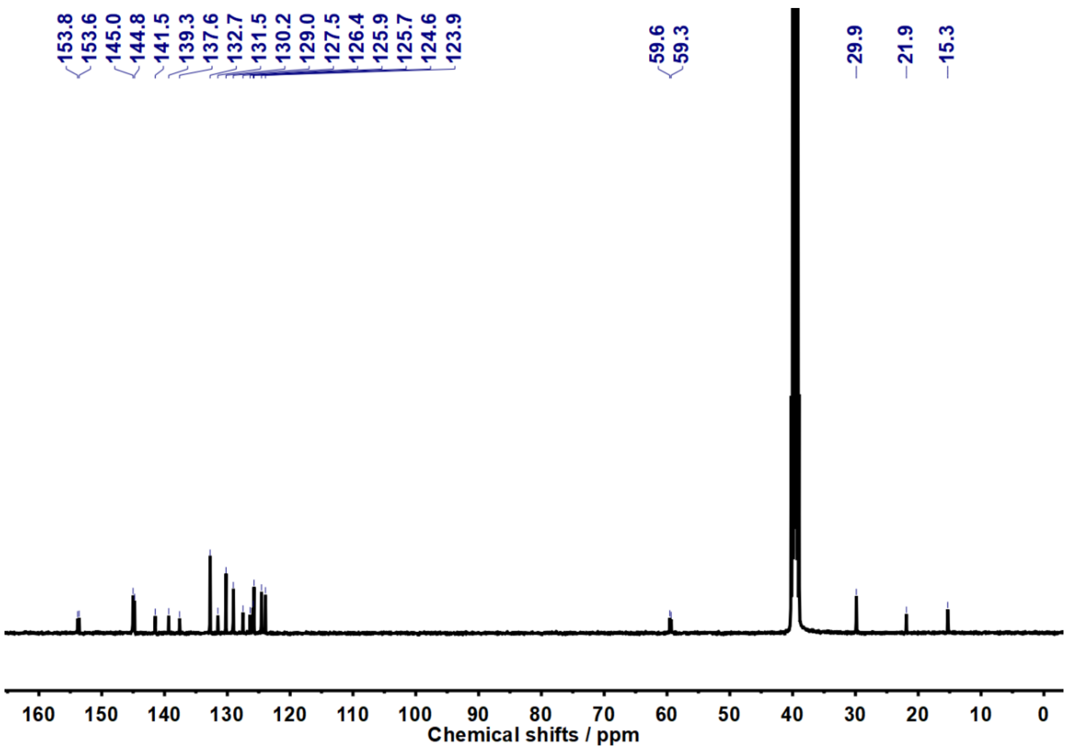

Figure S48. ^{13}C NMR spectrum (100 MHz, DMSO, 25 °C) of C5.

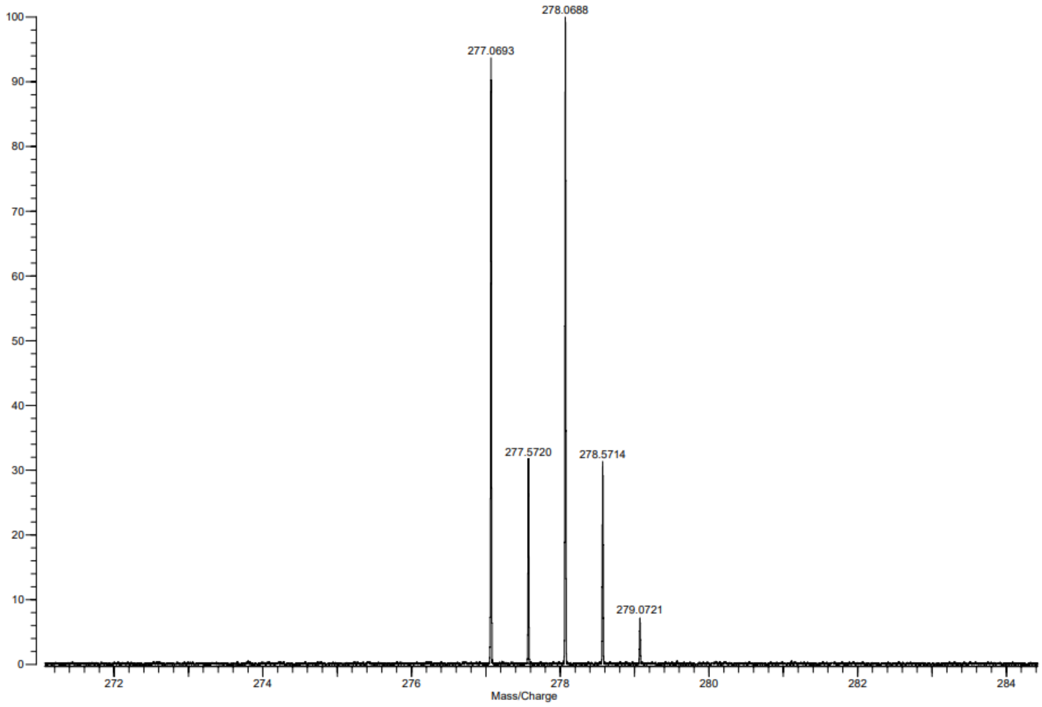

Figure S49. HR-MS of C5.

6. Computational details

All structures were pre-optimized with gau_xtb[2], followed by a further optimization using B3LYP functional[3] with DFT-D3(BJ) dispersion correction[4] in Gaussian 16 program[5]. A mixed basis set of SDD[6] for Br, 3-21G(d) for atoms in pre-optimized CB[8] and 6-31G(d) for other atoms. TDDFT[7] was used for calculating excited states. Computed structures were illustrated by CYLView.[8]

a)

b)

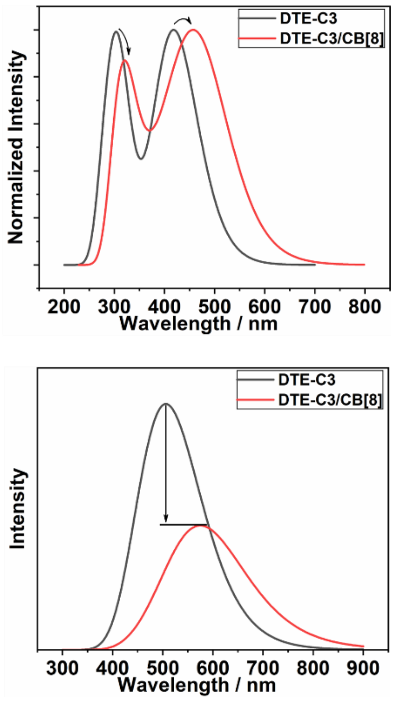

Figure S50. a) calculated absorption spectrum and b) calculated fluorescence spectrum. Noted that the calculated absorption spectrum shows similar bathochromic shift with experimental result upon adding CB[8], and fluorescence were quenched after the addition of CB[8].

7. Reference

1. X.-K. Ma, W. Zhang, Z. Liu, H. Zhang, B. Zhang, Y. Liu, *Adv. Mater.* ***2021***, *2007476*.

2. Lu, T., gau_xtb: A Gaussian interface for xtb code, http://sobereva.com/soft/gau_xtb (Aug. 20th, 2021)

3. (a) A. D. Becke, *J. Chem. Phys.* **1993**, *98*, 5648. (b) K. Raghavachari, *Theor. Chem. Acc.* **2000**, *103*, 361. (c) A. D. Becke, *J. Chem. Phys.* **1993**, *98*, 1372. (d) C. Lee,; W. Yang,; R. G. Parr, *Phys. Rev. B* **1988**, *37*, 785.

4. S. Grimme,; S. Ehrlich,; L. Goerigk, *J. Comput. Chem.* **2011**, 32, 1456.

5. M. J. Frisch, G. W. Trucks, H. B. Schlegel, G. E. Scuseria, M. A. Robb, J. R. Cheeseman, G. Scalmani, V. Barone, B. Mennucci, G. A. Petersson, H. Nakatsuji, M. Caricato, X. Li, H. P. Hratchian, A. F. Izmaylov, J. Bloino, G. Zheng, J. L. Sonnenberg, M. Hada, M. Ehara, K. Toyota, R. Fukuda, J. Hasegawa, M. Ishida, T. Nakajima, Y. Honda, O. Kitao, H. Nakai, T. Vreven, J. A. Montgomery, Jr., J. E. Peralta, F. Ogliaro, M. Bearpark, J. J. Heyd, E. Brothers, K. N. Kudin, V. N. Staroverov, T. Keith, R. Kobayashi, J. Normand, K. Raghavachari, A. Rendell, J. C. Burant, S. S. Iyengar, J. Tomasi, M. Cossi, N. Rega, J. M. Millam, M. Klene, J. E. Knox, J. B. Cross, V. Bakken, C. Adamo, J. Jaramillo, R. Gomperts, R. E. Stratmann, O. Yazyev, A. J. Austin, R. Cammi, C. Pomelli, J. W. Ochterski, R. L. Martin, K. Morokuma, V. G. Zakrzewski, G. A. Voth, P. Salvador, J. J. Dannenberg, S. Dapprich, A. D. Daniels, O. Farkas, J. B. Foresman, J. V. Ortiz, J. Cioslowski, and D. J. Fox, *Gaussian 09, Revision E.01.*, Gaussian, Inc., Wallingford CT, 2013.

6. D. Andrae,; U. Häuermann,; M. Dolg,; H. P. Stoll, , H. *Theor. Chem. Acc.* **1990**, *77*, 123.

7. (a) J. P. Stephens, J. J. Pan, F. J. Devlin, and J. R. Cheeseman. *In: J. Natural Prod.* **2008**, *71*, 285. (b) W. Liang, S. A. Fischer, M. J. Frisch, and X. Li. *In: J. Chem. Theory Comput.* **2011**, *7* 3540. (c) P. J. Lestrange, P. D. Nguyen, and X. Li. *In: J. Chem. Theory Comput.* 2015, *11* 2994.

8. Legault, C. Y. ***CYLview, 1.0b;*** Université de Sherbrooke, 2009 (http://www.cylview.org).
